# Potentials and pitfalls of transient in vitro reporter bioassays: interference by vector geometry and cytotoxicity in recombinant zebrafish cell lines

**DOI:** 10.1007/s00204-020-02783-6

**Published:** 2020-05-23

**Authors:** Sebastian Lungu-Mitea, Johan Lundqvist

**Affiliations:** Department of Biomedicine and Veterinary Public Health, Swedish University of Agricultural Sciences, Box 7028, SE-750 07 Uppsala, Sweden

**Keywords:** In vitro bioassays, Effect-based tools, Transient transfection, Cytotoxicity, Non-specific effects, Oxidative stress

## Abstract

The water framework directive re-evaluation proposes the integration of effect-based tools, increasing the need for alternative methods. Especially within aquatic toxicology, coverage of specific toxicity pathways is scarce, and most applications are based on mammalian or bacterial models, not reflecting realistic exposure scenarios. The use of transient reporter gene assays in cells from organisms of interest could be a quick and inexpensive solution. However, interference with cellular homeostasis may impact the system beyond the function of the manipulated gene and thus lead to non-specific results. We describe how varying vector geometry and different regulatory gene elements on plasmids used for transfection in zebrafish hepatocytes and embryonic fibroblasts may lead up to a tenfold difference in potency. Cells were transiently co-transfected with an Nrf2-responsive Firefly luciferase reporter plasmid and eight different Renilla luciferase normalization plasmids. Transfected cells were exposed to two different regimes (0.1–100 µM and 7.8–250 µM) of the oxidative stress-inducing compounds, sulforaphane, *tert*butylhydroquinone, and metazachlor. Nrf2 activity was measured in dual-luciferase assays. In parallel, cytotoxicity was assessed for different endpoints (energy metabolism, protein amount, membrane stability, and cell proliferation) in non-transfected cells and cells co-transfected with constructs of increasing size, to be used for normalization. Transfected cells were more susceptible to cytotoxicity in a vector size-dependent manner. Conclusively, we report that vector geometries (size, backbones, gene-regulatory units), cell line (tissue origin), applied transfection methods, and signal normalization may alter the sensitivity of reporter bioassays in a synergistic manner. Further, we propose that thorough bioassay design is needed to ensure reliability and regulatory acceptance.

**Electronic supplementary material:**

The online version of this article (10.1007/s00204-020-02783-6) contains supplementary material, which is available to authorized users.

## Introduction

Legislation on the manufacturing of chemicals to protect human health and the environment from adverse effects, such as the REACH (Registration, Evaluation, Authorization, and Restriction of Chemicals) directive or TSCA (Toxic Substances Control Act) reauthorization, have increased the demand in animal toxicity testing (Goldberg 2010; Hartung 2011). This scenario seems contradictory to the collective attempt to minimalize animal testing within the context of 3Rs (Russell and Burch 1959) and beyond (Lillicrap et al. 2016). Therefore, international regulatory agencies and stakeholders of the scientific community (NRC, EPA, ECVAM) have established new frameworks (e.g. “21st-century toxicology”) (NRC 2007; EURL-ECVAM 2014; Halder et al. 2014; US EPA 2016) which are promoting or demanding alternatives to in vivo tests.

Reporter gene assays in transfected mammalian cells for analyzing specific toxicity-related modes of action are valuable tools for research in many fields of toxicology (Zacharewski 1997; Ankley et al. 1998; Mueller 2004; Leusch and Snyder 2015) and an essential step stone in the adverse outcome pathway (AOP) concept (Ankley et al. 2010). However, this type of alternative approaches, focusing on molecular initiating events in important toxicity pathways, are still underrepresented within aquatic toxicology. Development of in vitro assays using fish cells has been proposed as a promising strategy to reduce and replace the use of fish in aquatic toxicity testing (Ankley et al. 2010; Halder et al. 2014; Worth et al. 2014). Such assays would allow high-throughput screening, application of omics technologies, and modeling approaches to risk assessment, such as conducted in ToxCast using mammalian cells (Judson et al. 2014). Notably, some fish cell cultures and fish embryo tests have emerged as useful alternative approaches in environmental toxicology (Garcia et al. 2016).

The European Water Framework Directive (WFD) (European Commission 2009) aims to achieve a good ecological and chemical status of surface water, mostly via chemical analysis of priority substances. Nevertheless, a multitude of anthropogenic substances and mixture effects are not assessed by this approach. To bridge the gap between chemical analysis and biomonitoring and also account for the unknown, the addition of a triad consisting of advanced chemical analysis (e.g., non-target screening), effect-based tools (bioanalysis/bioassays), and effect-directed analysis has been discussed (Altenburger et al. 2015) and has recently been recommended for the upcoming WFD revision (Brack et al. 2017, 2018, 2019). Originally, the WFD already lists a few in vivo bioassays as complementary methods (European Commission 2009), and suitable in vitro assays for the potential WFD revision were suggested (Wernersson et al. 2015). Most of the proposed effect-based tools are based on either mammalian cells or bacteria. To maximize the ecologic relevance for aquatic organisms, it is highly prioritized to develop effect-based tools, such as in vitro bioassays, based on fish cells (Lillicrap et al. 2016).

As a step towards this direction, we previously developed a transient reporter gene assay for analysis of oxidative stress in *D. rerio* fibroblast (ZF4) and liver (ZFL) cell lines (Lungu-Mitea et al. 2018), by measuring induction of the nuclear factor erythroid 2-related factor 2 (Nrf2), a major upstream regulator of ROS detoxification and metabolization (Itoh et al. 2004). The same principle assay design can be used for other molecular initiating events, allowing the investigation of diverse toxicity pathways in fish cells. With this approach, the use of in vitro assays based on fish cells has the potential to reduce the number of test animals.

However, it is noteworthy that the widespread assumption that either transient or stable transgene integration into a host preserves the genotypic, epigenetic, and phenotypic traits of the latter is not universally valid. Instead, transgenesis itself (via the used vectors, transfection reagents, gene cassettes for antibiotics, and patterns of expression) and experimental conditions (squelching, knockouts, chemical exposures, and culture conditions) are inflicting systemic stress. This may lead to an impact on the biological system beyond the function of the manipulated gene and thus to nonspecific effects (reviewed in Stepanenko and Heng 2017). Within toxicology, transgenic cell models are often used to monitor certain receptor activation, e.g., in the context of stress response and detoxification. For example, glucocorticoid receptor (GR)-based reporter assays of the pRL and pGL vector series were reported being problematic in the context of DNA transfection (Kushner et al. 1994; Dougherty and Sanders 2005), due to influencing the activity of major transcription factors and thereby competing with other receptor pathways for common response elements (Martino et al. 2004). Therefore, designing plasmid vectors to be applied in studies of steroid binding, cellular metabolism, and cellular stress defense pathways may be accompanied by certain challenges.

A detailed assessment of the previously named Nrf2 reporter assay seemed plausible, given that the oxidative stress response is potentially affected by cellular metabolism and the overall stress defense. Additionally, designing reporter gene assays for measuring oxidative stress can be problematic in the context of cytotoxicity, given that the role and effect of ROS changes include the whole range from physiological to pathological functions (Sharma et al. 2015). Noteworthy, a general induction of detoxifying mechanism at exposure concentrations close to those causing cytotoxicity was identified and termed the “cytotoxic burst” (Judson et al. 2016). Such induction of toxicity pathways is considered nonspecific, especially in terms of receptor-mediated toxicity. Given that rather small increases in ROS concentration are changing the response from signal transduction to induction of oxidative stress, and further apoptosis or necrosis (Redza-Dutordoir and Averill-Bates 2016), oxidative stress has to be assessed differently than receptor-mediated toxicity (Escher et al. 2012, 2018).

We hypothesize that the specific combination of used reporter and normalization vectors, in regard to inherent gene-regulatory units and plasmid geometry, has a crucial effect on cellular homeostasis and will influence the potency and reliability of transient reporter gene assays. To increase the sensitivity and reliability of the previously established transient Nrf2-responsive reporter gene assay, we performed an in-depth investigation of various parameters potentially influencing the induction of activity and the cytotoxicity of chemicals. Besides the primary Firefly luciferase reporter vector, a panel of Renilla luciferase normalization vectors was used in co-transfection, bearing different plasmid geometries (backbones and constitutive promoters). The results should be taken into consideration for the future design of reporter gene bioassays, their potency, and, beyond, their regulatory acceptance.

## Materials and methods

### Cell culture

#### Zebrafish fibroblast cell line ZF4

Embryonic zebrafish fibroblast cell line ZF4 (Driever and Rangini 1993) (CVCL_3275) was cultured in Dulbecco’s modified Eagle's medium/Ham’s Nutrient Mixture F-12 (DMEM:F12) containing phenol red (Gibco, Paisley, UK), supplemented with 10% (v/v) fetal bovine serum (Gibco, Paisley, UK), 1% (v/v) penicillin–streptomycin 100 U/mL (Gibco, Paisley, UK), 2.5 mM L-glutamine (Lonza, Basel, Swiss), 15 mM HEPES (Gibco, Paisley, UK), 0.5 mM sodium pyruvate (Sigma-Aldrich, Steinheim, Germany), and 1200 mg/L sodium bicarbonate (Gibco, Paisley, UK). The cells were cultured in a humidified environment at 28 °C and with 5% CO_2_. The cells were passaged weekly in a 1:10 ratio, using phosphate-buffered saline (PBS; at pH 7.4) (Medicago, Uppsala, Sweden) for washing and 0.25% (w/v) trypsin (Sigma-Aldrich, Steinheim, Germany) for detachment.

#### Zebrafish hepatocyte cell line ZFL

Zebrafish liver cell line ZFL (Ghosh et al. 1994; Eide et al. 2014) (CVCL_3276) was cultured in a medium consisting of 50% (v/v) Leibovitz’s L-15 (Sigma-Aldrich, Steinheim, Germany), 35% (v/v) Dulbecco’s modified Eagle's medium (Gibco, Paisley, UK), 15% (v/v) Ham’s Nutrient Mixture F-12 (Gibco, Paisley, UK), and phenol red. Additionally, 150 mg/L sodium bicarbonate (Gibco, Paisley, UK), 15 mM HEPES (Gibco, Paisley, UK), 10 µg/mL bovine insulin (Sigma-Aldrich, Steinheim, Germany), 50 ng/mL mouse EGF (Sigma-Aldrich, Steinheim, Germany), and 5% (v/v) fetal bovine serum (Gibco, Paisley, UK) were supplemented. The cells were cultured in a humidified environment at 28 °C and atmospheric CO_2_. The cells were passaged weekly in a 1:20 ratio, using PBS (pH 7.4) (Medicago, Uppsala, Sweden) for washing and 0.25% (w/v) trypsin-EDTA (Sigma-Aldrich, Steinheim, Germany) for detachment.

### Plasmids

The pGL4.37[luc2P/ARE/Hygro] plasmid was acquired from Promega (Madison, USA). The pGL4.37[luc2P/ARE/Hygro] vector consists of a pGL4 backbone including an ampicillin resistance gene, a gene for hygromycin resistance, and four copies of a Nrf2-sensitive antioxidant response element (ARE) driving transcription of the Firefly luciferase reporter gene luc2P (*Photinus pyralis*). Firefly luciferase was used as the primary reporter. Plasmids of the pRL series (Fig. S1 and Table S1) were acquired from Promega, Madison, USA. All plasmids consist of a pRL backbone including the cDNA (Rluc) encoding Renilla luciferase reporter gene (*Renilla reniformis*) and a specific constitutive promoter sequence (pRL-null: minimal promotor; pRL-TK: herpes simplex virus thymidine kinase promoter; pRL-SV40: simian virus 40 promotor; pRL-CMV: cytomegalovirus promotor). Plasmids of the pGL4.7x series (Fig. S1 and Table S1) were acquired from Promega, Madison, USA. All plasmids consist of a pGL4 backbone including the cDNA (hRluc) encoding modified, “humanized” Renilla luciferase reporter gene (*Renilla reniformis*) and a specific constitutive promotor sequence (pGL4.70: minimal promoter; pGL4.74: herpes simplex virus thymidine kinase promoter; pGL4.73: simian virus 40 promoter; pGL4.75: cytomegalovirus promoter). Renilla luciferases were used as control/normalization signals in the following dual reporter gene assays (DLR).

### Chemicals

The following known Nrf2 inducers were used for exposure studies: metazachlor (Met.) (CAS 67129-08-2), 99.5% purity (Dr. Ehrenstorfer GmbH, Augsburg, Germany); sulforaphane (SFN) (CAS 4478-93-7), 90% purity (Sigma-Aldrich, Steinheim, Germany); *tert*butylhydroquinone (tBHQ) (CAS 1948-33-0), 97% purity (Sigma-Aldrich, St. Louis, USA). 20 mM stock solutions of metazachlor and 100 µM tBHQ were prepared in 99% (v/v) EtOH and stored at  − 20 °C. A 50 mM stock of SFN in 99% (v/v) EtOH was prepared and stored at  − 80 °C.

### Handling, platting, transfection, and exposure

ZF4 and ZFL cells were seeded either into white, clear-bottom 96-well plates (Corning, New York, USA; for DLR experiments and ATP-viability assays), black, clear-bottom 96-well plates (Thermo Scientific Nunc., Roskilde, Denmark; for EdU-viability assay), or transparent 96-well plates (Corning, New York, USA; for MTS and BCA-viability assays) at a density of 2.5 × 10^5^ cells/mL for ZF4 and 1.5 × 10^5^ cells/mL for ZFL, in 100 µL/well. After 24 h of incubation, cells reached a confluency of about 80%.

Transfection was carried out in a 2 µg transfection reagent to 1 µg DNA ratio using FHD (Promega, Madison, USA) for ZF4, and XHP (Roche, Mannheim, Germany) for ZFL. Transfection optimization experiments were reported priorly (Lungu-Mitea et al. 2018). Transfection reaction was conducted as a co-transfection, using the pGL4.37[luc2P/ARE/Hygro] plasmid and specific plasmids of the pRLx and pGL4.7x series in a 10:1 reporter to control ratio (0.9 µg reporter plasmid, 0.1 µg control plasmid) for DLR experiments and viability assays in the specific combinations pGL4.37+pRL null/pGL4.70. To account for potential artifacts of co-transfection, ZF4 cells were also transfected solely with pGL4.37, pRL CMV, and pGL4.70, using 1 µg of plasmid per reaction.

After 24 h of post-transfection incubation, cells were exposed to Nrf2 inducers. Prepared stock solutions were further diluted using the cell type-specific nutrition medium supplemented with 5‰ (v/v) EtOH as a solvent. Seeded cells on 96-well plates were exposed in quadruplicate to increasing nominal concentrations (0.1 µM, 1 µM, 10 µM, 100 µM) of Nrf2 inducers tBHQ, SFN, and metazachlor for primary experiments, and to narrower titration steps for in-depth analysis (metazachlor: 7.8-250 µM; SFN: 0.31-10 µM; tBHQ: 3.12-100 µM) in 100 µL/well. Thereby, nominal concentrations of viability tests were always plated in triplicate. Double quadruplicates of 5‰ EtOH solvent–nutrition medium were used as controls. Passive lysis buffer (PLB; Promega, Madison, USA) and 10% (v/v) DMSO-containing exposure medium (Sigma-Aldrich, Steinheim, Germany) were used as positive controls in the viability tests.

### Dual reporter gene assay

Following exposure, cells were lysed in 20 µL PLB and quantitative Nrf2-dependent luminescence was measured via Dual-Luciferase^®^ Reporter Assay (DLR) (Promega, Madison, USA), according to the manufacturer’s protocol using an auto-injecting Infinite M1000 microplate reader (Tecan, Männedorf, Switzerland), following a flash luminescence protocol. The luciferase activity was expressed as fold change compared to the non-treated controls, both as normalized Firefly/Renilla readouts and single luciferase read-outs.

### Cell viability testing

To determine cytotoxic concentrations of the used compounds within the exposure range, cell viability was examined using various assays, covering diverse endpoints of cellular stress. Initial viability tests were conducted in parallel to the DLR experiments, using non-transfected cells, both for ZF4 and ZFL cell lines. Therefore, an MTS-based assay (see below) was applied. For further in-depth viability analyses, ZF4 cells were exposed to metazachlor in the range of 7.8–250 µM. To show the potential impact of transfection on viability, cells were either not transfected or transfected with pGL4.37 and pRL null or pGL4.70 plasmids, respectively.

#### MTS assay

MTS-based [3-(4,5-dimethylthiazol-2-yl)-5-(3-carboxymethoxyphenyl)-2-(4-sulfophenyl)-2H-tetrazolium] CellTiter 96® AQueous One Solution Cell Proliferation Assay (Promega, Madison, USA) was conducted in accordance with the manufacturer's protocol. Following exposure, 17% (v/v) MTS reagent was added to wells. After 2 h of incubation at 28 °C and a specific CO_2_ atmosphere, formazan product turnover absorbance was measured at 490 nm using an Infinite M1000 microplate reader (Tecan, Männedorf, Switzerland). A mean blank control (in triplicate; no cells, nutrition medium plus substrate only) was subtracted from all raw values. Relative effects on cell viability were calculated in relation to the vehicle controls.

#### ATP/LDH-multiplex assay

The CellTiter-Glo® Luminescent Cell Viability Assay (Promega, Madison, USA) for quantification of ATP present in viable cells and the CytoTox-ONE™ Homogeneous Membrane Integrity Assay (Promega, Madison, USA) for the measurement of lactate dehydrogenase (LDH) release from cells with damaged membranes were multiplexed according to the manufacturer's protocol and prior publications (Farfan et al. 2005), with minor alterations. After exposure, plates were shaken for 2 min on an orbital shaker and 50 µL of every well was transferred to black 96-well plates (Thermo Scientific Nunc., Roskilde, Denmark), already containing 50 µL/well CytoTox-ONE™ resazurin substrate mix (50% (v/v)). Following 10 min of incubation at room temperature (RT) on an orbital shaker in darkness, 25 µL/well stop solution was added. In parallel, 50 µL/well Ultra-Glo™ Recombinant Luciferase substrate (50% (v/v)) was added to the original 96-well plates and incubated for 1 h at RT on an orbital shaker in darkness. Fluorescence (560 nm ex./590 nm em.) or luminescence (1 s integration time for glow luminescence), for LDH or ATP measurement, respectively, was recorded on an Infinite M1000 microplate reader (Tecan, Männedorf, Switzerland). A mean blank control (in triplicate; no cells, nutrition medium plus substrate only) was subtracted from all raw values. Relative effects on cell viability were calculated in relation to the vehicle control for the ATP assay and in relation to the lysis positive control for the LDH assay.

#### BCA assay

Bicinchoninic Acid Protein Assay (BCA, Sigma-Aldrich, St. Louis, USA) was conducted following the manufacturer's protocol with minor alterations. After exposure, the medium was discharged and cells of every well were lysed in 20 µL PLB at RT for 15 min on an orbital shaker. 180 µL of BCA reagent was added to every well, and plates were agitated shortly and incubated for 20 min at 60 °C. After cooling down for 15 min at RT, absorbance was measured at 562 nm using an Infinite M1000 microplate reader (Tecan, Männedorf, Switzerland). A mean blank control (in triplicate; no cells, nutrition medium plus substrate only) was subtracted from all raw values. The effects were calculated in relation to the vehicle control.

#### EdU assay

The Click-iT® EdU Microplate Assay was used for the determination of cell proliferation. The EdU (5-ethynyl-2'-deoxyuridine) substrate contains a nucleoside analog of thymidine and is incorporated into DNA during active DNA synthesis which can be detected fluorometrically. Handling and exposure of cells were conducted as described above with the difference that 1 µM EdU was additionally supplemented to the exposure medium. The following steps were exactly performed according to the manufacturer’s protocol. Fluorescence (568 nm ex./585 nm em.) was read on an Infinite M1000 microplate reader (Tecan, Männedorf, Switzerland). A mean blank control (in triplicate; no cells, nutrition medium plus substrate only) was subtracted from all raw values. The response in cell proliferation was normalized to the vehicle control.

### Statistical analyses

**Fig. 1:**
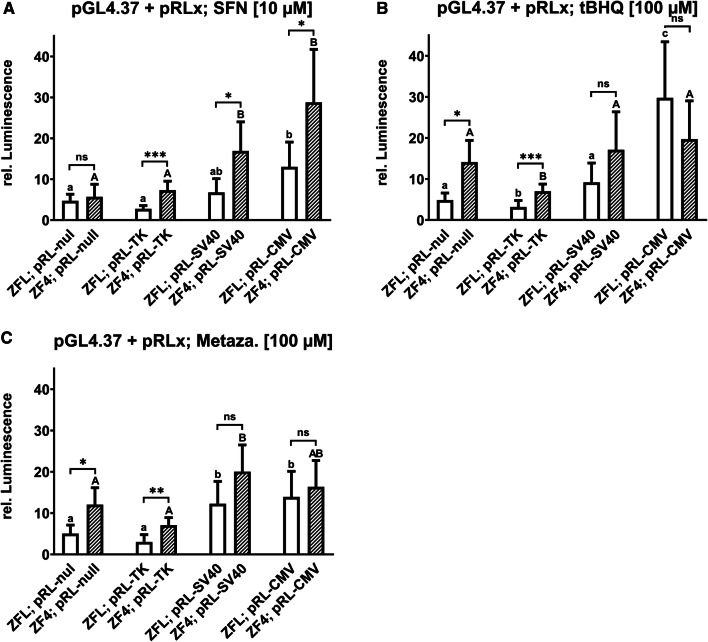
Effects on luminescence measured in the zebrafish cell lines ZFL and ZF4 treated with selected nominal concentrations of sulforaphane (**a**; SFN), *tert*butylhydroquinone (**b**; tBHQ), and metazachlor (**c**). Luminescence corresponds to quantitative Nrf2 activation measured via DLR assay in cells co-transfected with pGL4.37 and the specifically depicted normalization vectors. Normalized values are depicted as white bars for ZFL and striped bars for ZF4. Each bar represents the mean (experimental units *n* = 3; observational units *N* = 12) including SD. Asterisks indicate significance between different cell lines for identical transfection setups, tested in a two-way ANOVA mixed model with Tukey’s post hoc test (ns *P* > 0.05; *P < 0.05, **P < 0.01, ***P < 0.001). Lowercase letters indicate statistically significant differences between transfection setups in the ZFL cell line (*P* < 0.05). Uppercase letters indicate a statistically significant difference between transfection setups in the ZF4 cell line (*P* < 0.05). Numerical means are illustrated in Tab. S2

**Fig. 2:**
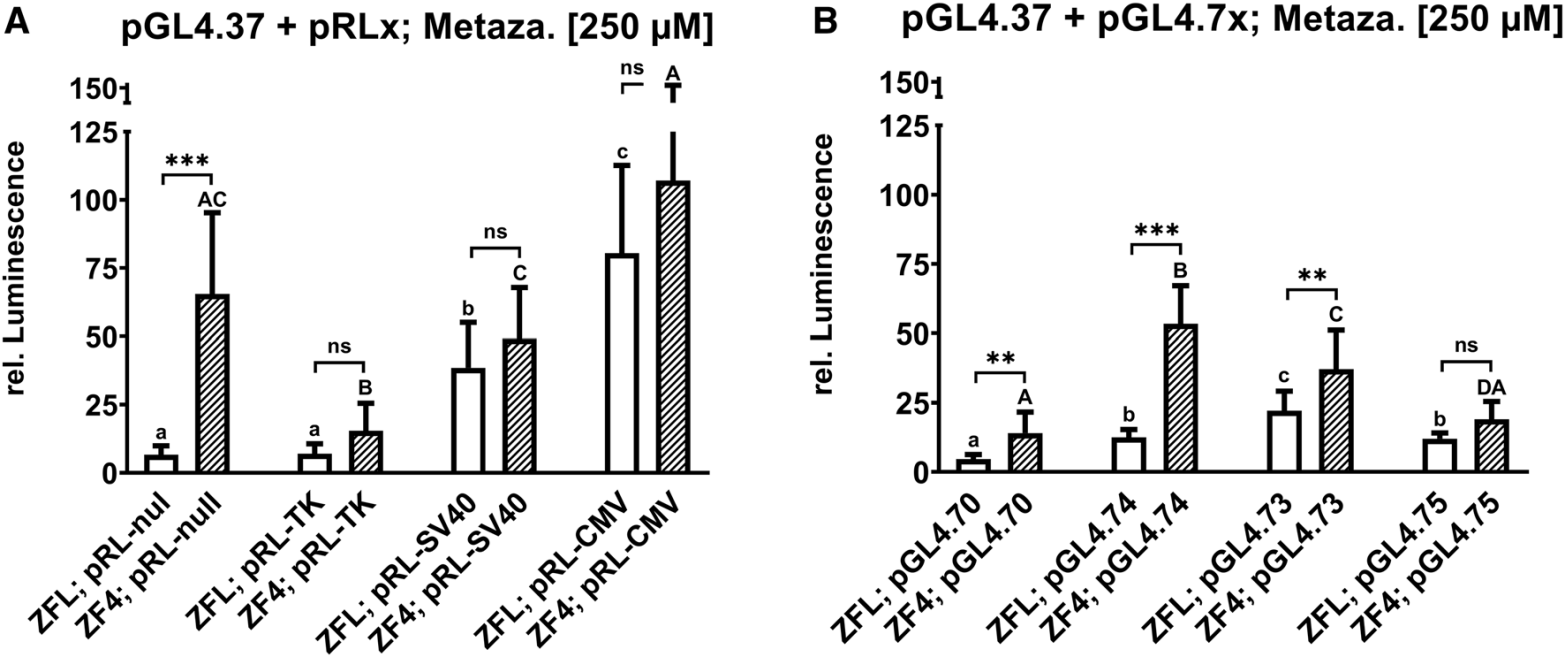
Effects on luminescence measured in the zebrafish cell lines ZFL and ZF4 treated with 250 µM metazachlor. Luminescence corresponds to quantitative Nrf2 activation measured via DLR assay in cells co-transfected with pGL4.37 and the specifically depicted normalization vectors of the pRLx (**a**) and pGL4.7x (**b**) series. Normalized values are depicted as white bars for ZFL and striped bars for ZF4. Each bar represents the mean (experimental units *n* = 3; observational units *N* = 12) including SD. Asterisks indicate significance between different cell lines for identical transfection setups, tested in a two-way ANOVA mixed model with Tukey’s post hoc test (ns *P* > 0.05; *P < 0.05, **P < 0.01, ***P < 0.001). Lowercase letters indicate statistically significant differences between transfection setups in the ZFL cell line (*P* < 0.05). Uppercase letters indicate a statistically significant difference between transfection setups in the ZF4 cell line (*P* < 0.05). Numerical means are illustrated in Tab. S3

**Fig. 3:**
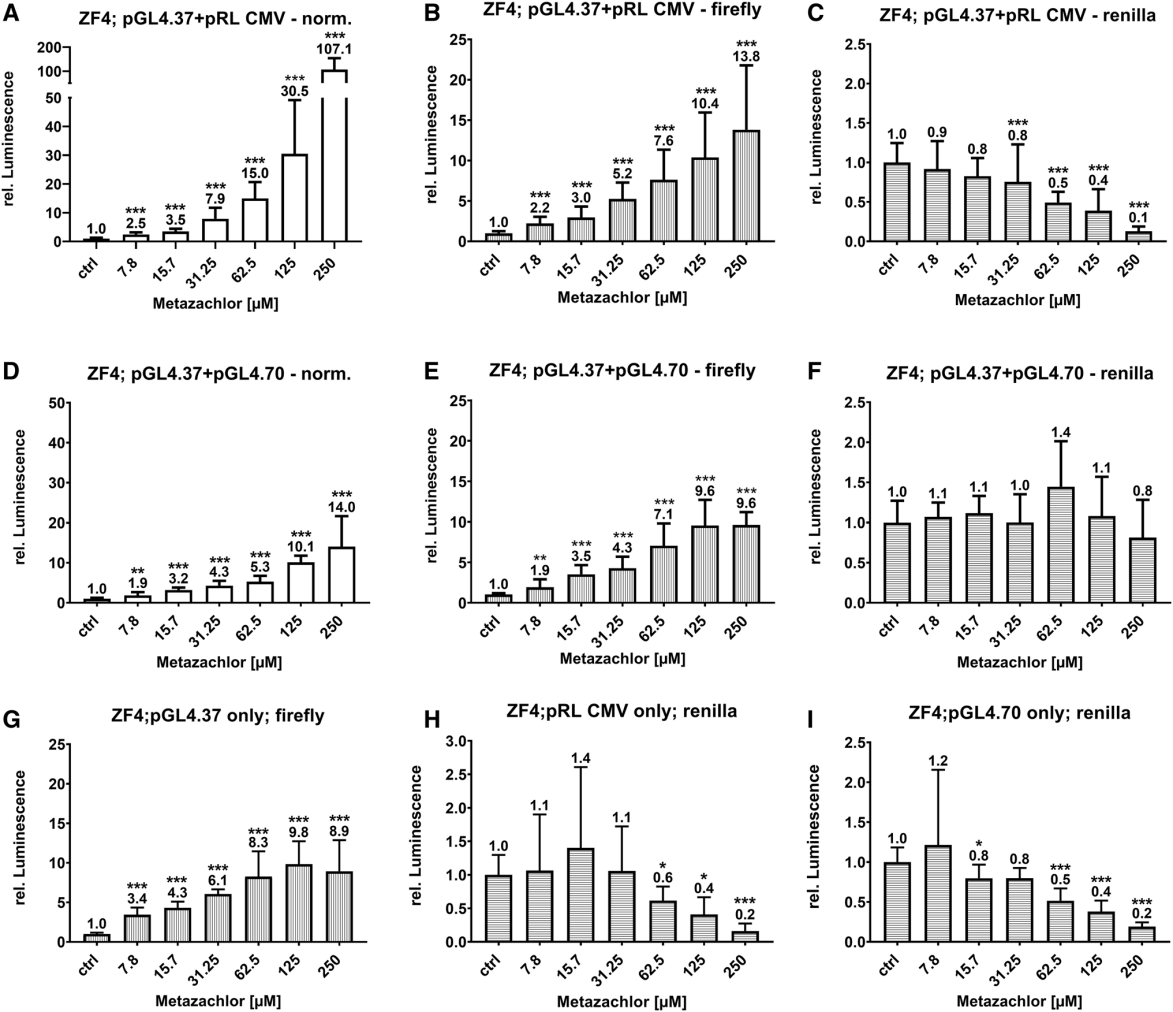
Effects on luminescence measured in the zebrafish cell line ZF4 treated with metazachlor. Luminescence corresponds to quantitative Nrf2 activation measured via DLR assay in cells co-transfected with pGL4.37 (a–f) and the normalization vectors pRL-CMV (a–c) and pGL4.70 (d–f). To uncover co-transfection artifacts, the specific plasmids were also solely transfected (g–i). Normalized values are depicted as white bars, Firefly luciferase read-outs as gray bars with vertical stripes, and Renilla luciferase read-outs as gray bars with horizontal stripes. Each bar represents the mean (experimental units *n* = 3–4; observational units *N* = 10–16) including SD. Numerical means are depicted on top of bars. Asterisks indicate significance tested in a two-way ANOVA mixed model with Dunnett's post hoc test (*P < 0.05, **P < 0.01, ***P < 0.001)

**Fig. 4:**
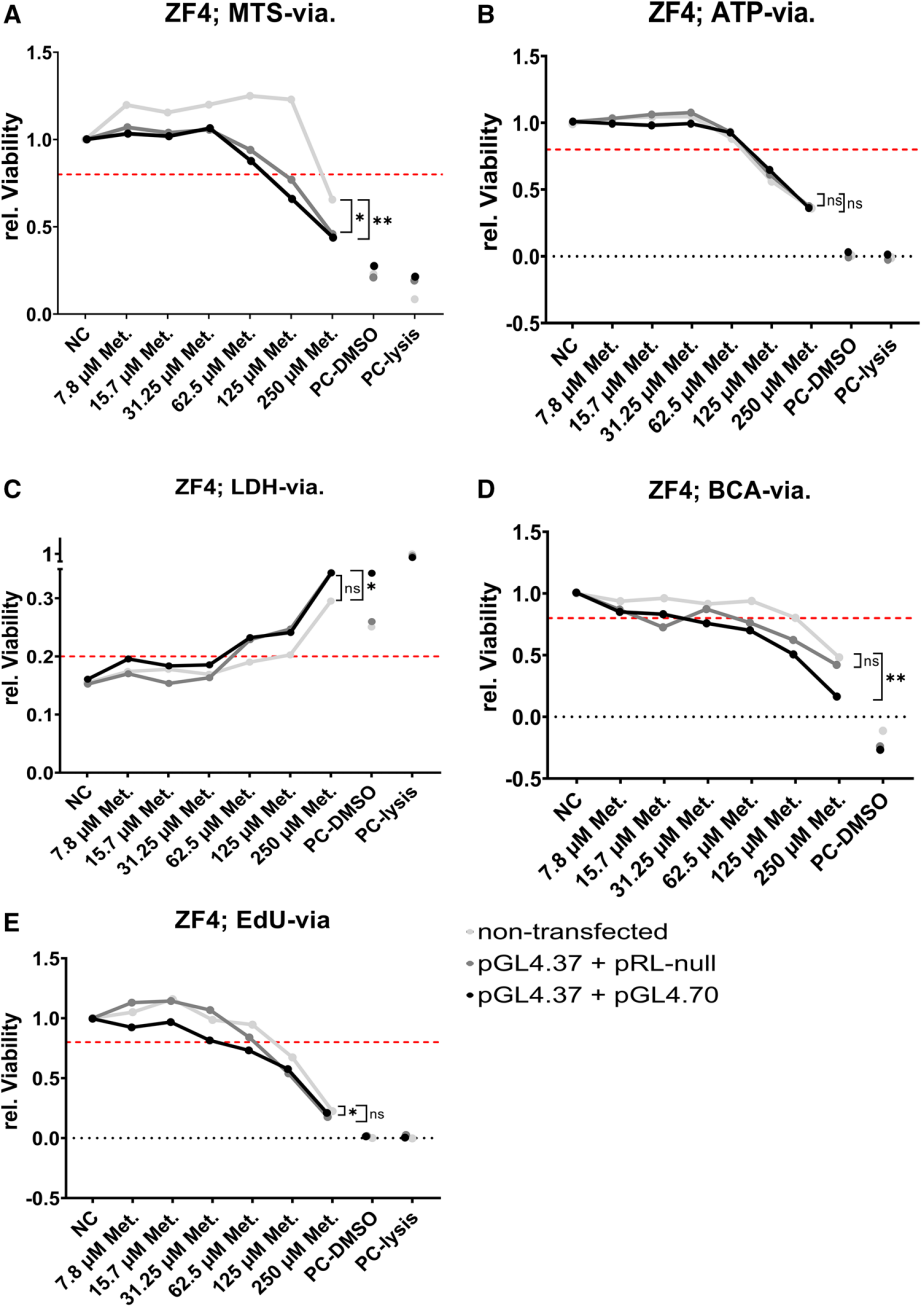
Effects on various viability end points (dots connected by lines) measured in the zebrafish cell line ZF4 treated with metazachlor (Met.). End points quantified are NAPDH metabolism via the MTS assay (**a**), ATP turnover (**b**) and LDH release (**c**) via the ATP/LDH multiplex assay, total protein amount via the BCA assay (**d**), and cell proliferation via the EdU assay (**e**). A solvent control was used as negative control (NC). Cellular lysis buffer (PC-lysis) and 10% (v/v) of DMSO in nutrition medium (PC-DMSO) were used as positive controls. Initial values were normalized to the NC or PC (**c**), respectively. A threshold value of 0.8 or 0.2 (for LDH) was considered biologically relevant/significant (dotted red line). Each point represents the mean (experimental units *n* = 3–4; observational units *N* = 9–12). Asterisks indicate significance of main transfection effect tested via two-way ANOVA, followed by Holm–Sidak’s post hoc test (ns *P* > 0.05; *P < 0.05, **P < 0.01, ***P < 0.001). For every end point, firstly, non-transfected cells were exposed (light gray dots), and, secondly, cells were co-transfected with pGL4.37 and normalization vectors of increasing size, pRL null (3320 nt; dark gray dots) and pGL4.70 (3522 nt; black dots). For details and further statistics, see also Fig. S15 and Tab. S6

Results from the DLR assays and the viability assays were processed using *R* and GraphPad Prism 8 (GraphPad Software, La Jolla, USA). Graphs and illustrations were designed using GraphPad Prism 8. For both approaches, data of three or four experiments (experimental unit *n* = 3–4), performed in either triplicate (viability assays) or quadruplicate (DLR) for each concentration, were pooled, giving a total population size for every exposure group of 9–16 (observational unit *N* = 9–16). Background (blanks) was subtracted and data were normalized against the vehicle control, giving fold induction as a final output. Normality was tested by Shapiro–Wilk and Kolmogorov–Smirnov tests (both significance level alpha = 0.05) and analyzed graphically by normal qq plot. Non-normal data were log transformed and re-analyzed. Given normality, statistical significance of the concentration-effect factor (transformed output data) was assessed via a mixed-effects model two-way ANOVA (Lazic 2010), followed by Dunnett’s post hoc test (for comparison vs*.* control) or Tukey’s post hoc test (for comparison in between groups). Thereby, transformed output data were considered as a fixed factor, whereas experiments were considered as a random factor within the model, to account for inter-experimental variation. A *P* value < 0.05 was considered statistically significant (Figs. 1, 2, 3). Residuals were graphically analyzed by quantile–quantile plot (actual vs*.* predicted residuals), homoscedasticity plot (absolute residuals vs*.* fitted), and residual plot (residuals vs. fitted) to ensure ANOVA criteria were met. For the statistical analysis of the transfection setup effects (mean column effects) means of single experiments were pooled (experimental unit *n* = observational unit *N* = 3–4) and significant differences between mean column effects were analyzed via two-way ANOVA followed by Holm–Sidak’s post hoc test (Fig. 4 and Table 1). An assessment of residuals was conducted as stated previously. Beyond statistical significance, for all viability tests, a threshold of 80% as compared to the negative control (corresponding 0.8) was determined as biologically significant and marked with a dotted line within all respective graphs.

**Table 1:** Two-way ANOVA results of overall main effect (transfection setup) of exposure patterns depicted in Fig. 3 (ns *P* > 0.05; *P < 0.05, **P < 0.01, ***P < 0.001)

Holm–Sidak’s test	Mean diff.	Significance	Adjusted *p* value	Output
A vs. D	− 0.3292	***	< 0.0001	Normalized
G vs. E	0.0818	ns	0.1417	Firefly Lum.
G vs. B	0.0629	ns	0.2394	
B vs E.	− 0.0190	ns	0.6422	
I vs. H	− 0.0521	ns	0.3603	Renilla Lum.
I vs. F	− 0.2244	***	< 0.0001	
I vs. C	0.0597	ns	0.3603	
H vs. F	− 0.1724	**	0.0017	
H vs. C	0.1118	ns	0.0544	
F vs. C	0.2841	***	< 0.0001	

## Results and discussion

Previously, we described a reporter gene assay to measure oxidative stress via Nrf2 induction in transiently transfected zebrafish cell lines (Lungu-Mitea et al. 2018), which could be a tool for aquatic organism-centered bioassay screening of environmental samples. Today, most effect-based screening activities rely on mammalian or bacterial reporter-gene assays (Macova et al. 2011; Escher et al. 2012; Wernersson et al. 2015; Neale et al. 2015, 2017; Rosenmai et al. 2018); using cells from aquatic organisms would, therefore, increase the relevance of the results. However, a multitude of cis- and trans-acting effects potentially alternate the outcome of transient transcription (Stepanenko and Heng 2017). In this study, we have used transiently transfected zebrafish cell lines to test different combinations of an Nrf2-responsive Firefly luciferase reporter with eight Renilla luciferase normalization vectors (Fig. S1 and Tab. S1). We monitored various endpoints of cytotoxicity to investigate how luciferase induction and reporter activity are impacted by the plasmid geometry (gene-regulatory elements/promoters and backbones), and how cytotoxicity further affects the outcome.

### Impact of constitutive promoters of the normalization vector on luciferase induction

To investigate if the constitutive promoter of the normalization vector impacts the luciferase induction, ZFL and ZF4 cells were transfected with a Firefly luciferase reporter fused to an Nrf2-induced ARE enhancer (pGL4.37[luc2P/ARE/Hygro]) in combination with different pRLx Renilla luciferase normalization vectors, containing the CMV, SV40, TK, and null (minP) constitutive promoters of different strength (Fig. S1 and Tab. S1). These promoters were shown to be active in various fish cell lines (Isa and Shima 1987; Inoue et al. 1990; Friedenreich and Schartl 1990; Liu et al. 1990; Bearzotti et al. 1992; Bétancourt et al. 1993; Ruiz et al. 2008; Martinez-Lopez et al. 2013). Transiently transfected cells were first exposed to increasing nominal concentrations (0.1, 1, 10, and 100 µM) of known Nrf2 inducers sulforaphane (SFN), *tert*butylhydroquinone (tBHQ), and metazachlor (Met.). We observed major differences in signal induction between the cell lines depending on which constitutive promoter was used (Fig. 1), at all concentrations of Nrf2 inducers. Concentration–response patterns for each cell line are presented in Fig. S3 (ZFL) and S4 (ZF4). For both cell lines, the general trend of potency of induction was TK ≤ null < SV40 < CMV. Cell viability of non-transfected cells measured by the MTS assay is presented in Fig. S5. Non-cytotoxic concentrations inducing the highest activity (Fig. S3 and S4) were selected for further statistical examination and are presented as follows. In Fig. 1, the statistical significance between the applied transfection scenarios at one selected concentration is indicated by uppercase letters for ZF4 and lowercase letters for ZFL. Induction was generally higher in the ZF4 cell line (Fig. 1; Tab. S2), with the exception of tBHQ at 100 µM in ZFL using the CMV promoter (Fig. 1b). Normalized luciferase signal induction, in dependency of the used constructs for transfection, differed significantly between cell lines in at least one exposure scenario (pR-null: Fig. 1b+c; pRL-TK: Fig. 1–c; pRL-SV40: Fig. 1a; pRL-CMV; Fig. 1a). Interestingly, patterns of induction potency after exposure to known Nrf2 inducers resemble the Renilla background signal illustrated in Fig. S2C+D. Hence, potency might be influenced by promoters in use.

One could expect less overall interference by secondary factors for weaker promoters, thus giving stronger signals after normalization to transfection conditions. Unexpectedly, we observed the opposite. Nevertheless, higher interference by backbone-only co-transfected, “empty” vectors was reported (Hofman et al. 2000), e.g., via minP transactivation by basic helix-loop-helix (bHLH) transcription factors Hand1 and Hand2 (Hong et al. 2002). Except for TK, the promoter strengths that were reported in mammalian cell lines could also be confirmed here in zebrafish cell lines (for RLU (raw luminescence unit) values within solvent controls see Fig. S2 and Tab. S4), indicating sufficient evolutionary conservation of transcription factors. Spurious expression deriving from Renilla normalization plasmids has previously been reported for all promoters tested here (reviewed in Shifera and Hardin 2010) within different cellular models. Especially the TK-promoter element seems to be notorious for spurious up- and downregulation by secondary factors (Shifera and Hardin 2010). In accordance with this, in the ZF4 cell line, both TK-containing plasmids pRL-TK and pGL4.74 show elevated Firefly background RLUs within the controls when compared to the other promoters (Fig. S2F). Thus, using TK promoters might induce the Nrf2 response independently of oxidative stress or chemical exposure and further lower the total inducibility. Cryptic binding sites of various steroid/thyroid/retinoid superfamily nuclear receptors were identified on either the plasmid backbones, promoter sequences, or the Renilla luciferase gene sequence itself of the TK promoter-containing vectors (Everett and Crabb 1999; Ibrahim et al. 2000; Zhang et al. 2003). Especially, interference with common transcription factors was repeatedly reported for TK-bearing plasmids (by specific protein-1 (sp1) (Osborne and Tonissen 2002), Nurr1 (Matuszyk et al. 2002), GATA-4/6 (Ho and Strauss 2004), and muscle-specific transcription factor (skNAC) (Sims et al. 2003), among others). Given that many of the named factors are involved in cellular development, homeostasis, and response to stress, a spurious expression of the pRL-TK vector under circumstances of cellular stress seems plausible. A similar case of spurious Renilla luciferase induction was reported for an androgen-responsive reporter assay (Mulholland et al. 2004), in dependency of the used promoters, the co-transfected transgenes, and the used cell line. Here, the authors postulated that the effect is unlikely to originate at the transcript or protein level of Renilla luciferase, but a result of the specific plasmids in use.

### Impact of vector backbones on luciferase induction

To investigate if the vector backbone alters the luciferase induction, we compared the effects of metazachlor on cells transfected with plasmids based on two different backbones, given that metazachlor displays the best dynamic exposure range of the used Nrf2-inducing compounds and no cytotoxicity-related non-monotonic concentration–response patterns were recorded. It has been suggested to use vectors with identical backbones in co-transfection, to decrease possible trans-effects of transcription co-factors (Nejepinska et al. 2014). Vectors of the pGL4.7x series bearing the same constitutive promoters as the pRLx series (see Fig. S1 and Tab. S1) were co-transfected with pGL4.37 in parallel to the pRLx series in both ZFL (Fig. S6 and Fig. S7) and ZF4 (Fig. S8 and Fig. S9) cell lines and exposed to increasing nominal concentrations (7.8, 15.7, 31.25, 62.5, 125, and 250 µM) of metazachlor. Viability in non-transfected cells (Fig. S14) was measured in parallel as above, using the MTS assay. For the pRLx series, patterns are comparable to the results discussed in section 3.1. In terms of induced luminescence, the potency of induction is TK ≤ null < SV40 < CMV (Fig. 2a). The pattern observed in the pGL4.7x series was different from the pattern observed in the pRLx series for both ZFL and ZF4 (Fig. 2b), with null < CMV < TK < SV40 for ZFL and null ≤ CMV < SV40 < TK for ZF4. When looking at Renilla RLUs (Fig. S2c+d), it becomes apparent that basic induction differs in the pattern (null < TK < SV40 < CMV) and strength, even statistically for every transfection setup. Nevertheless, induction patterns are not conserved throughout exposure (Fig. 2b), as opposed to the pRLx series. Further, it seems that co-transfection with the pRLx series results in generally stronger signals than with the pGL4.7x series (Fig. 2), except for pGL4.74. Also, ZF4 shows generally higher induction than ZFL (Fig. 2 and Tab. S3), differing significantly for pRL-null, pGL4.70, pGL4.73, and pGL4.74. A possible explanation could be the basic Firefly RLUs, which are more constant for ZFL (Fig. S2e) but also generally higher (Tab. S5), if omitting artificially upregulated activity of the TK promoter-bearing vectors. Lowest basic Firefly RLUs were recorded for ZF4, especially in the transfection setups including pRL-CMV, pGL4.70, and pGL4.75, with the TK-bearing constructs differing even statistically significant (Fig. S2f), as already discussed above. Additionally, setups that gave high basic Firefly RLUs (pRL-TK, pRL-SV40, and pGL4.74 for ZF4) also depicted irregularly high Renilla induction patterns within higher metazachlor exposure concentrations (Fig. S8f+i). Hence, the general overall basic induction of the signal within the ZFL line might also lead to a generally lower total inducibility of the normalized results.

Out of all the combinations tested, co-transfection with pRL-CMV and pGL4.70 in ZF4 cells is depicted here (Fig. 3a–f) in more detail, since these resulted in the most opposing reporter activity within one cell line and did not show artificial upregulation or high standard deviation in Firefly RLUs, such as TK- and SV-40-bearing vectors. ZF4 showed a more pronounced response regarding induction, and comparably low basic Firefly RLUs within the solvent controls. Remarkably, the normalized induction increases almost tenfold when comparing the two normalization plasmids pRL-CMV (107.1) and pGL4.70 (14), which is also statistically significant as an overall effect (Table 1). Non-normalized values for Firefly (Fig. 3b+e; Fig. S6–S9) and Renilla (Fig. 3c+f; Fig. S6–S9) measurements are also plotted as induced luminescence to investigate the different setups more accurately. The Firefly induction is generally higher for ZF4 with a maximum fold increase of around 15 in the highest effect concentrations when co-transfected with pRLx vectors and around 10 with pGL4.7x vectors (Fig. S8+9). For ZFL, Firefly induction only reaches a maximum of up to approximately fivefold and differences between the backbone series are not as pronounced as in ZF4 (pattern ZF4_pRLx > ZF4_pGL4.7x > ZFL_pRLx ≥ ZFL_pGL4.7x) (Fig. S6+7). Hence, the data correspond to the observations made above. Regarding the Renilla measurements, it may be hypothesized that the stronger basic RLU induction has a negative impact on the maximum induction of Firefly (Fig. S2d), as particularly encountered for pGL4.75 (Fig. 2b; Fig. S2d; Table S4; Fig. S7j–l), thus impeding a dynamic response in reaction to the stressor. Nevertheless, as described above, using weak promoters does not necessarily result in the strongest overall signals. Figure 3 shows the normalized reporter activity (panel a and d) for the two setups which gave the most perpendicular signals, as well as the Firefly (panels b and e) and Renilla (panels C and F) measurements separately. For Firefly, patterns are comparable between the two setups with induction not differing statistically (Table 1). However, the patterns for Renilla activity differ statistically (Table 1) between the two setups. Whereas pRL-CMV (Fig. 3c) shows a decreasing Renilla activity with increasing metazachlor concentrations, pGL4.70 (Fig. 3f) remains stable with increasing metazachlor concentrations. We conclude that the reduction in Renilla values in the pGL4.37+pRL-CMV setup was the main reason for the observed differences in sensitivity after normalization. When transfecting only single vectors, Firefly patterns and mean induction remain comparable to co-transfected setups (Fig. 3g compared to 3b and 3e) and also do not differ statistically (Table 1). Interestingly, the Renilla read-out patterns and values for pRL-CMV are quite comparable between single (Fig. 3h) and co-transfection (Fig. 3c). On the other hand, for pGL4.70 (Fig. 3i), patterns between single and co-transfection differ statistically, showing a concentration-dependent decrease in Renilla activity with increasing metazachlor concentrations (Table 1). It may be concluded that co-transfection itself alternates the transcription and translation of the used constructs and may impact the normalized result. To prove that the encountered patterns are not specifically due to metazachlor exposure, but a general outcome in response to stressors and Nrf2 inducers, the same experimental setups were applied once for SFN and tBHQ in the ZF4 cell line (see Fig. S10–S13), with similar results.

The introduction of foreign, potent transcriptional activators into eukaryotic cells was reported to suppress the transcript of a co-transfected gene. This phenomenon is termed “squelching” (Natesan et al. 1997) and describes the competition between gene-regulatory elements for general transcription factors, coactivators, and the general transcription/translation machinery (Simon et al. 2015). Given that these resources are limited within a single cell, this competition may lead to overall reduced transcription levels. Thereby, squelching is primarily encountered for episomal target genes, but obsolete for genes within the cellular chromatin. The total amount of artificially introduced expression vectors may exceed the capacity of the cellular transcriptional/translational machinery, and therefore competition of the reporter construct and the co-transfected normalization construct may appear in a size- and potency-dependent manner (Hofman et al. 2000; Hu et al. 2002). Nevertheless, the extent of squelching was also reported to be cell line dependent (Adam et al. 1996), due to different physiological prerequisites of tissue origin, given that networks of cellular regulation are cell type and even cell cycle stage specific (Dumont et al. 2002). The squelching phenomenon could describe many effects encountered here. The pGL4.7x gave a generally lower reporter signal and generally higher Renilla RLU values after co-transfection than the pRLx series. pGL4 vectors are of the newest generation and codon optimized for better transcription (Paguio et al. 2005). The reporter vector pGL4.37 is also based on these backbones. When comparing overall Firefly luciferase induction patterns for ZF4 co-transfected with pRLx (Fig. S8) to pGL4.7x (Fig. S9), a general higher induction is visible. Additionally, for ZF4, Firefly induction decreases with increasing strength of promoters on the normalization vectors. Whereas this effect is only marginal for the pRLx series, it is evident for pGL4.7x, with induction values dropping from approximately tenfold maximal induction for pGL4.70 to maximal fivefold induction for pGL4.75 (Fig. S9b+k). Regarding ZFL, Firefly induction is neither impacted by the used backbone nor by used constitutive promoters, but generally lower than for ZF4, as mentioned above. This may have physiological reasons due to tissue origin or result from an alternate transfection protocol and will be discussed later. Potential explanations for these observed effects could be that using the same backbone for normalization in combination with a stronger constitutive promoter may occupy resources of the overall transcription/translation machinery and lead to lower induction of the reporter gene.

Alternatively, differences in reporter gene induction may be explained by varying backbone geometry and additional regulatory elements. Former reports conducted in the carp epithelial cell line (EPC) claimed that the presence of functional introns is highly beneficial for transgene expression, especially when compared to mammalian counterparts (Friedenreich and Schartl 1990; Bétancourt et al. 1993). The pRLx series is still bearing such functional introns, whereas these have been obliterated in the pGL4x series (see also Fig. S1) (Paguio et al. 2005), given that constructs are mainly optimized for mammalian transgenesis. Therefore, the induction of Renilla normalization reporter on the pRLx series may respond more dynamically to stressors and thus give better results.

### Potential link between cytotoxicity and effects on the normalization vector

As we concluded from the previous section, variation in normalized reporter gene activity was mainly caused by differences in Renilla luciferase activity of the normalization plasmid. These differences may be caused by increasing cytotoxicity, which is not entirely reflected by the cell viability assay in use. The MTS assay only measures one end point for cell viability. So, to get a broader picture of cytotoxicity, we applied additional assays targeting different biological endpoints of cell viability.

MTS viability tests with non-transfected cells conducted in parallel to the DLR assays showed cytotoxicity by significance or threshold definition at 250 µM metazachlor for ZFL and 125 and 250 µM metazachlor for ZF4 (Fig. S14a+b). The cytotoxicity of the highest metazachlor exposures could also be observed as a decrease in the Firefly activity in the ZFL cells (Fig. S6 and Fig. S7; panels b, e, h, k). Similarly, we observed a decrease in the Firefly activity after exposure to high concentrations of SFN in ZF4 (Fig. S10 and Fig. S11; panels b, e, h, k), which is probably due to cytotoxicity. However, Firefly values were decreasing already at concentrations that do not induce cytotoxicity, as indicated by the MTS assay (Fig. S14d).

A possible explanation for this is that the MTS viability test, which is based on NADPH turnover, might not be sensitive enough in general or in specific for the ZFL line to detect the cytotoxicity causing the decrease in Renilla activity. MTS results have previously been reported as being misleading (reviewed in Stepanenko and Dmitrenko 2015), and potential artifacts can be caused by various nutrition medium components, such as serum, antioxidants, or vitamins (Zhang and Cox 1996; Huang et al. 2004; Funk et al. 2007). Beyond that, interference by oxidative stress is possible. NADPH is involved in many response pathways regarding oxidative stress (Hayes and Dinkova-Kostova 2014), so decreasing levels in the presence of radicals may result in interference with the MTS assay. As a response to oxidative stress, glutathione S-transferase is induced. The latter was reported to reduce MTS substrate in vitro, leading to high background levels (York et al. 1998). Given the perspective that potential oxidative stress should be examined here, relying solely on MTS viability data would be critical. Therefore, a battery of viability assays, covering diverse endpoints of cellular stress (Fig. 4; Fig. S15; Tab. S6), was applied to ZF4 cells exposed to identical concentrations as in the DLR assays. This was done mainly to test the combined effect of co-transfection and exposure, to account for induced stress correctly, to investigate patterns of Renilla luciferase normalization readouts in co-transfection setups, and to back postulations made above. Noteworthy, cytotoxicity tests that are standardly conducted in parallel to reporter gene assays use non-transfected cells due to handling and economic reasons. To prove if the standard approach is scientifically correct, exposure was applied to non-transfected cells, and cells co-transfected with the pGL4.37 reporter vector and pRL null/pGL4.70 normalization vectors, bearing different backbone lengths (3320 nt. and 3522 nt, respectively).

When comparing the effects of metazachlor on cell viability of non-transfected ZF4 with the effects on transfected cells, we found that, in general, metazachlor induced cytotoxicity at lower concentrations in the transfected cells compared to the non-transfected cells (Fig. 4), which is the case for all measured endpoints except ATP (Fig. 4b). Further, the overall effect of transfection on cell viability was statistically significant for all assays except for the ATP assay. Thereby, the transfection setup using the larger pGL4.70 construct in co-transfection was statistically significantly different from non-transfected cells for the LDH, BCA, and EdU assays (Fig. 4c–e), and even statistically significant for both transfection setups for the MTS assay (Fig. 4a). Interestingly, mitochondria-dependent metabolic assays, such as MTS and ATP (Fig. 4a+b; Fig. S15a–f; Tab. S6), showed lower sensitivity to metazachlor than assays of alternate endpoints. We found that the BCA (Fig. 4d; Fig. S15j–l) and EdU assays (Fig. 4e; Fig. S15m–o) were the most sensitive. Notably, the decrease in Renilla activity is partly more severe than the decrease in relative viability even for the most sensitive endpoints (e.g., Fig. S8l compared to Fig. S15l+o). Interference with luciferase turnover may occur at a lower level of biological organization, most likely during transcription or translation. Therefore, deregulation of cellular homeostasis may be initiated at lower concentrations than measured in the viability endpoints. Thus, Renilla luciferase turnover may be regarded as a more sensitive endpoint of cellular stress than the standard viability tests at use.

Notably, it was reported that transfection induces immune response (Jacobsen et al. 2009), since it partly mimics a viral infection by the production of foreign RNA (Terenzi et al. 1999). Also, luciferases were reported to be inhibited by IFNs in a vector mass-specific manner via post-transcriptional mechanisms (Ghazawi et al. 2005). As depicted here, the vector size-specific induction of increased cellular stress in co-transfection may, therefore, potentiate the final response to a stressor. Interestingly, anti-proliferative effects of IFNs treatment were reported to be underestimated by tetrazolium-based viability assays, such as the MTS (Jabbar et al. 1989; Marionnet et al. 1997), also potentially explaining the lower sensitivity encountered here.

### Impact of transfection conditions and tissue origin on luciferase induction

As described above in regard to Firefly induction, besides potentially inhibitory effects of the pGL4.7x series, basic Nrf2 activity is higher or at least more stable for the ZFL cell line (Fig. S2a+e), thus possibly leading to lower inducibility. Especially, if artificially upregulated constructs (TK promoter bearing vectors) or constructs with high standard deviation in vehicle control Firefly RLUs (pRL-SV40) are omitted, it becomes evident that for the ZFL cell line basic Firefly RLUs are statistically higher than their ZF4 counterparts (Tab. S5). Tissue origin may be a plausible reason, since nose, gill, and liver have previously been identified as Nrf2 and downstream gene induction hot-spots in zebrafish (Nakajima et al. 2011). A possible alternate explanation is the different transfection reagents used. FHD (used for ZF4) was shown to be a potent reagent and induces only minimal amounts of toxicity, stress, and immune response (Jacobsen et al. 2009; Yalvac et al. 2009; Kim and Eberwine 2010; Yamano et al. 2010; Antczak et al. 2014; Lungu-Mitea et al. 2018). To our knowledge, no evaluation of these parameters has ever been conducted regarding the XHP reagent (used for ZFL). Cellular stress and induction of foreign DNA via transfection will initiate an immune response, interferon release, and activation of PKC and MAPK pathways. All these have been reported to interfere with cellular metabolism (Smith 2001; Jacobsen et al. 2009), transfection/translation homeostasis (Simon et al. 2015), or the used transfection vectors per se (Terenzi et al. 1999; Shifera and Hardin 2009, 2010). Beyond that, Nrf2 might not only be activated by oxidative stress-triggered release from its inhibitor Keap1, but also via phosphorylation by MAPK and PKC pathways (Bryan et al. 2013). Increased stress and immune response scenario induced by transfection would, therefore, lead to a higher Nrf2 activity within the solvent control as well, without any exposure to stressors, thus preventing a more dynamic response. We encountered this phenomenon by recording generally higher Firefly RLUs in the ZFL cell line for constructs that are not artificially upregulated (Fig. S2e and Tab. S5). Additionally, interferons, namely interferon alpha (IFNα) and interferon beta (IFNβ), were reported to inhibit Renilla luciferase expression from the pRL-TK vector (Ghazawi et al. 2005). According to this, the pRL-TK vector showed the lowest Renilla RLUs in both cell lines (Fig. S2c+d), and Firefly RLUs were highly up-regulated for TK-bearing plasmids in the ZF4 cell line (Fig. S2f). However, since we did not specifically look into an immune system response and potential activation of the MAPK and PKC pathways, these statements remain tentative and need to be backed in future studies.

### Luciferase induction is dependent on multiple experimental parameters

Potential sources of artifacts and spurious expression of reporters are genome integration and potential lesions (Smith 2001), squelching and resource limitations of transcriptional and translational machinery (Natesan et al. 1997; Simon et al. 2015), posttranscriptional interference via RNAi (Nejepinska et al. 2012), and immune response inhibition of transgene translation initiation by protein kinase C (PKC) and interferons (IFNs) due to non-native DNA (Nejepinska et al. 2014). Additionally, it has been reported that luciferase reporters tend to be inhibited by small molecules, either directly via competition for required substrate, or indirectly via enzyme denaturation or photonic processes (Auld et al. 2008a), further by enzyme stabilization and signal overestimation (Auld et al. 2008b; Thorne et al. 2010b), or by compound aggregation (Thorne et al. 2010a). In this study, we observed differences in signal induction when using varying vector geometries, such as used regulatory elements and vector size, cell line tissue or species origin, applied transfection methods, and signal normalization. Our results indicate that co-transfection itself can alternate the transcription and translation of the used constructs and may lead to potential inhibition or spurious overexpression within a specific cellular system under stress. Therefore, phenotypic responses after transfection are not only a result of single above-stated elements, but also a combination of various mechanisms. Hence, the complex setup of established reporter assays implies numerous influencing factors. Thus, results need to be assessed and interpreted cautiously. As a result, precautionary measures need to be taken in plasmid vector design, such as applied here, with rigorous viability testing of diverse endpoints, to display weak points (“pitfalls”) and potentials in regulatory frameworks.

## Conclusion

As postulated, our results indicate that plasmid geometry and gene-regulatory units have an effect on the potential outcome and potency of the reporter gene assay after co-transfection. We showed that promoter strength, as previously reported for mammalian cell lines, could be confirmed in zebrafish cell lines, except for the pRL-TK vector, indicating sufficient conservation of transcription factors. Thereby, TK-bearing plasmids seemed to be spuriously regulated in the cell lines used in this study. Further, differences in normalized luciferase signal induction were a result of the applied normalization vectors, specifically their constitutive promotors and backbones. The ZF4 cell line gave a stronger response to Nrf2-regulated oxidative stress than the ZFL cell line. Possible explanations for this difference in sensitivity could either be because of tissue specificity or transfection conditions. Also, co-transfection with the pRLx series resulted in generally stronger signals than with the pGL4.7x series. pRLx induction patterns were conserved throughout the exposure, whereas for pGL4.7x, they were not. Out of all combinations tested, in the ZF4 cell line, the Nrf2-responsive Firefly reporter vector pGL4.37, together with the Renilla normalization vector pRL-CMV, gave the strongest normalized reporter activity, which was up to tenfold higher than weaker combinations recorded. Within the same cellular context, Firefly inductions were stable across treatments, both for single and dual transfections. Thus, the Renilla values and specific normalization led to different outcomes in sensitivity. By applying a battery of viability test, covering diverse end points of cellular stress, we were able to prove that the transfection procedure itself increases cellular stress in a vector size-dependent manner. Further, these results affirmed that the most potent combination of co-transfected reporter and normalization vectors was, in fact, not the result of spurious inhibition, but a realistic depiction of actual Nrf2 signal induction in the context of increasing cellular stress and cell death. Nevertheless, it must be mentioned that co-transfection itself can alter the cellular environment, which can influence the biological pathway studied. Given that the final signal measured will always be a result of combined mechanisms, it is important to take precautionary decisions in plasmid vector design, to display weak points of the artificial system, and overcome intrinsic faults of the methodology. Thereby, reporter gene assays can be a potent tool for high-throughput screening of environmental samples, and these may acquire regulatory acceptance if designed, assessed, and applied properly.

## Electronic supplementary material

Below is the link to the electronic supplementary material.

Supplementary file1 (PDF 6149 kb)

## Data Availability

All data generated or analyzed during this study are included in this published article (and its supplementary files). Raw data are available from the corresponding author upon request.
