# Supplementary material for: Potentials and pitfalls of transient in vitro reporter bioassays: interference by vector geometry and cytotoxicity in recombinant zebrafish cell lines
Source: Arch Toxicol. 2020 May 23;94(8):2769–84. doi: 10.1007/s00204-020-02783-6 (PMC7395025; doi:10.1007/s00204-020-02783-6)
Supplement: Supplementary file 1 — Supplementary file1 (PDF 6149 kb) [file 204_2020_2783_MOESM1_ESM.pdf]

Potentials and pitfalls of transient *in vitro* reporter bioassays

Interference by vector geometry and cytotoxicity in recombinant zebrafish cell lines

Supplementary Material

Archives of Toxicology

Sebastian Lungu-Mitea*[a] and Johan Lundqvist[b]

Department of Biomedicine and Veterinary Public Health, Swedish University of Agricultural Sciences, Box 7028, SE-750 07 Uppsala, Sweden

* Corresponding author:

Sebastian Lungu-Mitea

E-mail: sebastian.lungu@slu.se

[a] https://orcid.org/0000-0001-8192-9134

[b] https://orcid.org/0000-0001-5693-9007

Index

Statistical analyses of supplementary material 4

Fig. S1/Tab. S1: illustration and table of applied vectors 5

Tab. S2: highest measured rel. luminescence induction (± SD) for ZFL and ZF4 cell lines, co-transfected with pGL4.37 and pRLx 6

Tab. S3: highest measured rel. luminescence induction for ZFL and ZF4 cell lines, co-transfected with pGL4.37 and pRLx/pGL4.7x respectively at an exposure concentration of 250 µM metazachlor 6

Fig. S2: RLU values within solvent controls 7-8

Tab. S4: RLU value range (± SD) within solvent controls after co-transfection 8

Tab. S5: Unpaired t-test of ZF4 vs ZFL mean Firefly RLU values within solvent controls 8

Fig. S3: Effects of SFN, tBHQ, and metazachlor on luminescence measured in the zebrafish cell line ZFL (primary exposure regime), co-transfection: pGL4.37+pRLx 9

Fig. S4: Effects of SFN, tBHQ, and metazachlor on luminescence measured in the zebrafish cell line ZF4 (primary exposure regime), co-transfection: pGL4.37+pRLx 10

Fig. S5: Effects of SFN, tBHQ, and metazachlor on viability (MTS) measured in non-transfected zebrafish cell lines ZFL and ZF4 (primary exposure regime) 11

Fig. S6: Effects of metazachlor on luminescence measured in the zebrafish cell line ZFL (secondary exposure regime), co-transfection: pGL4.37+pRLx 12

Fig. S7: Effects of metazachlor on luminescence measured in the zebrafish cell line ZFL (secondary exposure regime), co-transfection: pGL4.37+pGL4.7x 13

Fig. S8: Effects of metazachlor on luminescence measured in the zebrafish cell line ZF4 (secondary exposure regime), co-transfection: pGL4.37+pRLx 14

Fig. S9: Effects of metazachlor on luminescence measured in the zebrafish cell line ZF4 (secondary exposure regime), co-transfection: pGL4.37+pGL4.7x 15

Fig. S10: Effects of SFN on luminescence measured in the zebrafish cell line ZF4 (secondary exposure regime), co-transfection: pGL4.37+pRLx 16

Fig. S11: Effects of SFN on luminescence measured in the zebrafish cell line ZF4 (secondary exposure regime), co-transfection: pGL4.37+ pGL4.7x 17

Fig. S12: Effects of tBHQ on luminescence measured in the zebrafish cell line ZF4 (secondary exposure regime), co-transfection: pGL4.37+pRLx 18

Fig. S13: Effects of tBHQ on luminescence measured in the zebrafish cell line ZF4 (secondary exposure regime), co-transfection: pGL4.37+ pGL4.7x 19

Fig. S14: Effects of SFN, tBHQ, and metazachlor on viability (MTS) measured in non-transfected zebrafish cell lines ZFL and ZF4 (secondary exposure regime) 20

Fig. S15: Effects of metazachlor on various viability endpoints measured in the zebrafish cell line ZF4 (secondary exposure regime), co-transfection with constructs of increasing size 21-22

Tab. S6: threshold-based (tre.) and statistically (stat.) derived LOECs in [µM] after metazachlor exposure in ZF4 cells using assays for diverse cellular viability endpoints 22

Statistical analyses of supplementary material

Data were handled and analyzed, as described in detail within section 2.7 of the main article. For fig. S3-4, S6-9, S14A+B, S15: mixed-effects model two-way ANOVA followed by Dunnett's post-hoc test with fixed concentration-effect factor (transformed output data) and random experiment factor (observational unit N = 9-16 for every concentration-group tested; $P < 0.05$ was considered statistically significant). Alternatively, for experiments only conducted once (experimental unit n = 1; observational unit N = 3-4), significant differences ($P < 0.05$) between tested groups were analyzed via one-way ANOVA followed by Dunnett's post-hoc test (fig. S10-13, S14C+D). In case normality could not be achieved by transformation, means of single experiments were pooled (experimental unit n = observational unit N = 3-4) and significant differences ($P < 0.05$) between the control and the exposure groups were analyzed via a non-parametric one-way ANOVA (Kruskal-Wallis test) followed by Dunn's post-hoc test (fig. S5). An assessment of residuals was conducted, as stated in the main article. A 0.8 threshold of biological significance was added to viability data, as described in the main article.

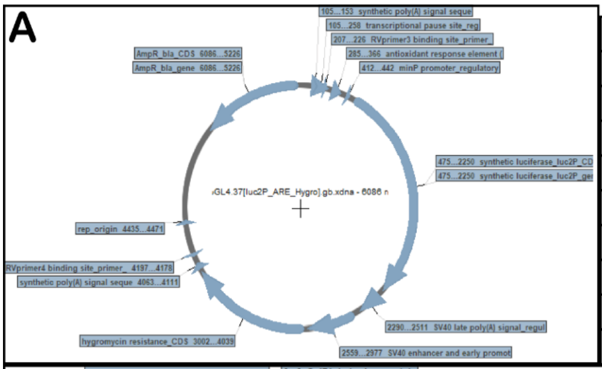

Plasmid vector	[promoter/enhancer]	Reporter
pGL4.37 (A)	[ARE]	Firefly
pGL4.70 (B)	[minP]	Renilla
pGL4.74 (C)	[TK]	Renilla
pGL4.73 (D)	[SV40]	Renilla
pGL4.75 (E)	[CMV]	Renilla
pRL-null (F)	[minP]	Renilla
pRL-TK (G)	[TK]	Renilla
pRL-SV40 (H)	[SV40]	Renilla
pRL-CMV (I)	[CMV]	Renilla

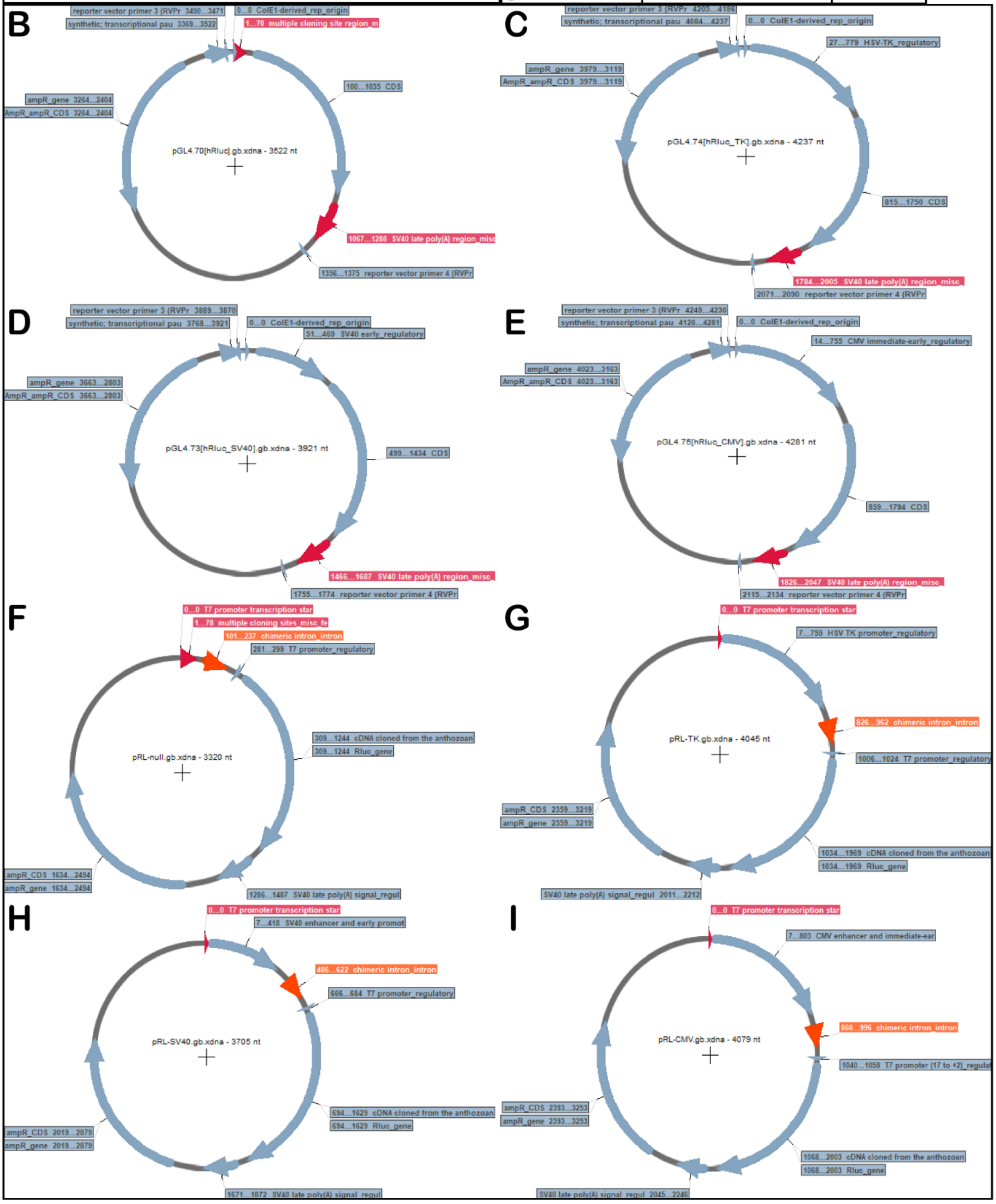

Fig. S1/Tab. S1: Illustration and table of applied vectors. Plasmid maps were generated from GenBank repository files using Serial Cloner 2.6.

Tab. S2: Highest measured rel. luminescence induction (± SD) for ZFL and ZF4 cell lines, co-transfected with pGL4.37 and pRLx; for respective graphical illustrations, see fig. 1, fig. S3, and fig. S4.

	SFN [10 µM]		tBHQ [100 µM]		Met. [100 µM]	
	**ZFL**	**ZF4**	**ZFL**	**ZF4**	**ZFL**	**ZF4**
**pRL-null**	4.7 (± 1.5)	5.7 (± 2.9)	4.9 (± 1.6)	14.1 (± 5.1)	5 (± 2)	12 (± 3.9)
**pRL-TK**	2.8 (± 0.8)	7.3 (± 2.1)	3.2 (± 1.5)	7 (± 1.7)	3 (± 1.7)	7.1 (1.8)
**pRL-SV40**	6.8 (± 3.2)	16.9 (± 6.8)	9.2 (± 4.6)	17.1 (± 9)	12.3 (± 5.2)	20.1 (±6.2)
**pRL-CMV**	13 (± 5.8)	28.8 (± 12.5)	29.8 (± 13.2)	19. 7 (± 8.9)	13.9 (± 5.9)	16.4 (± 6.1)

Tab. S3: Highest measured rel. luminescence induction for ZFL and ZF4 cell lines, co-transfected with pGL4.37 and pRLx/pGL4.7x respectively at an exposure concentration of 250 µM metazachlor; for graphical illustrations see fig. 2 and fig. S6 to fig. S9.

	**pRL-null**	**pRL-TK**	**pRL-SV40**	**pRL-CMV**	**pGL4.70**	**pGL4.74**	**pGL4.73**	**pGL4.75**
**ZFL**	6.7 (± 3.1)	7 (± 3.5)	38.5 (± 15.9)	80.5 (± 30.7)	4.6 (± 1.6)	12.5 (± 2.7)	22.1 (± 6.8)	12 (±2)
**ZF4**	65.4 (± 28.5)	15.5 (± 9.8)	49.1 (±17.9)	107.1 (± 45)	14 (± 7.3)	53.3 (± 13.2)	37.1 (± 13.4)	19 (±6.2)

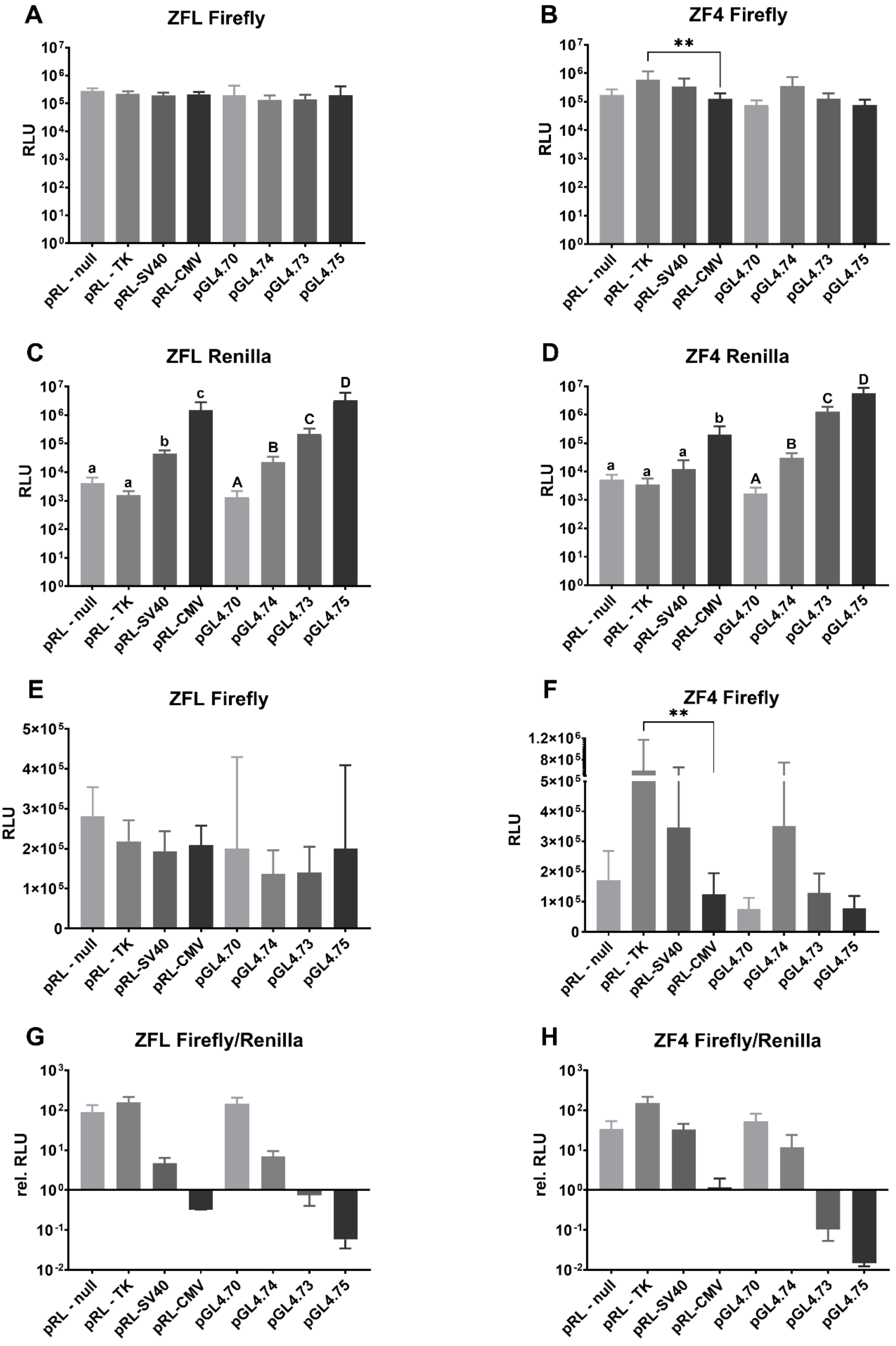

Fig. S2: RLU values within solvent controls for ZFL (A, C, E, G) or ZF4 (B, D, F, H) cell-lines, co-transfected with pGL4.37 and the respectively depicted normalization vectors, recorded by DLR. RLUs correspond to Nrf2 background activation. Each bar represents the mean (see tab. S2 for experimental and observational units), including SD. Significance testing (one-way

ANOVA with Tukey's post-hoc test) was only conducted within plasmid backbone groups (pRLx or pGL4.7x). Asterisks indicate significance tested for Firefly induction (**P < 0.01). Lowercase letters indicate significance of Renilla induction within the pRLx backbone group (P < 0.05). Uppercase letters indicate significance of Renilla induction within the pGL4.7x backbone group (P < 0.05). Illustrated are Firefly RLUs (log-scale, A and B), Renilla RLUs (log-scale, C and D), Firefly RLUs (linear scale, E and F), and relative Firefly/Renilla RLUs (log-scale, G and H).

Tab. S4: RLU value range (± SD) within solvent controls after co-transfection of pGL4.37 and the respective normalization vectors, recorded by DLR, as exponentials 10^x = E+x; n = experimental units; N = observational units

	ZFL			ZF4		
	**Firefly**	**Renilla**	**Relative**	**Firefly**	**Renilla**	**Relative**
**pRL-null**	2.81E+05 ± 6.85E+04; n=9; N=54	4.10E+03 ± 2.26E+03; n=9; N=54	8.95E+01 ± 4.25E+01; n=9; N=54	1.72E+05 ± 9.15E+04; n=10; N=60	5.32E+03 ± 2.30E+03; n=10; N=60	3.39E+01 ± 1.85E+01; n=10; N=60
**pRL-TK**	2.19E+05 ± 4.92E+04; n=9; N=54	1.58E+03 ± 5.56E+02; n=9; N=54	1.56E+02 ± 5.54E+01; n=9; N=54	5.99E+05 ± 5.39E+05; n=9; N=54	3.48E+03 ± 2.00E+03; n=9; N=54	1.53E+02 ± 6.15E+01; n=9; N=54
**pRL-SV40**	1.93E+05 ± 4.80E+04; n=9; N=54	4.47E+04 ± 1.22E+04; n=9; N=54	4.74E+00 ± 1.59E+00; n=9; N=54	3.46E+05 ± 2.97E+05; n=10; N=60	1.25E+04 ± 1.16E+04; n=10; N=60	3.33E+01 ± 1.16E+01; n=10; N=60
**pRL-CMV**	2.09E+05 ± 4.66E+04; n=11; N=66	1.48E+06 ± 1.29E+06; n=11; N=66	3.26E-01 ± 3.34E-01; n=11; N=66	1.25E+05 ± 6.65E+04; n=11; N=66	1.97E+05 ± 1.90E+05; n=11; N=66	1.16E+00 ± 7.66E-01; n=11; N=66
**pGL4.70**	2.01E+05 ± 2.16E+05; n=9; N=54	1.33E+03 ± 8.17E+02; n=9; N=54	1.44E+02 ± 5.75E+01; n=9; N=54	7.54E+04 ± 3.52E+04; n=7; N=42	1.70E+03 ± 9.37E+02; n=7; N=42	5.28E+01 ± 2.75E+01; n=7; N=42
**pGL4.74**	1.37E+05 ± 5.27E+04; n=5; N=30	2.24E+04 ± 1.07E+04; n=5; N=30	7.02E+00 ± 2.23E+00; n=5; N=30	3.51E+05 ± 3.64E+05; n=7; N=40	3.10E+04 ± 1.20E+04; n=7; N=40	1.16E+01 ± 1.15E+01; n=7; N=40
**pGL4.73**	1.40E+05 ± 6.12E+04; n=10; N=54	2.14E+05 ± 1.14E+05; n=10; N=54	7.42E-01 ± 3.21E-01; n=10; N=54	1.29E+05 ± 6.05E+04; n=8; N=42	1.29E+06 ± 5.62E+05; n=8; N=42	1.04E-01 ± 4.71E-02; n=8; N=42
**pGL4.75**	2.00E+05 ± 2.00E+05; n=11; N=60	3.29E+06 ± 2.74E+06; n=11; N=60	5.76E-02 ± 2.18E-02; n=11; N=60	7.77E+04 ± 3.83E+04; n=7; N=42	5.68E+06 ± 2.84E+06; n=7; N=42	1.47E-02 ± 2.23E-03; n=7; N=42

Tab. S5: Unpaired t-test of ZF4 vs ZFL mean Firefly RLU values within solvent controls, co-transfected with pGL4.37 and the respectively named normalization vectors; according to fig. S2 E+F (ns P > 0.1; 'P < 0.1; * P < 0.05, ** P < 0.01, *** P < 0.001)

Unpaired t-test	Mean diff. ± SEM	Significance	*P*-value
pRL-null	0.2064 ± 0.09035	*	0.0385
pRL-TK	-0.2581 ± 0.1404	'	0.0858
pRL-SV40	-0.1426 ± 0.1172	ns	0.2403
pRL-CMV	0.2880 ± 0.09326	**	0.0058
pGL4.70	0.2734 ± 0.1023	*	0.0192
pGL4.74	-0.1843 ± 0.2459	ns	0.4709
pGL4.73	0.07453 ± 0.1156	ns	0.5282
pGL4.75	0.2933 ± 0.1656	'	0.0958

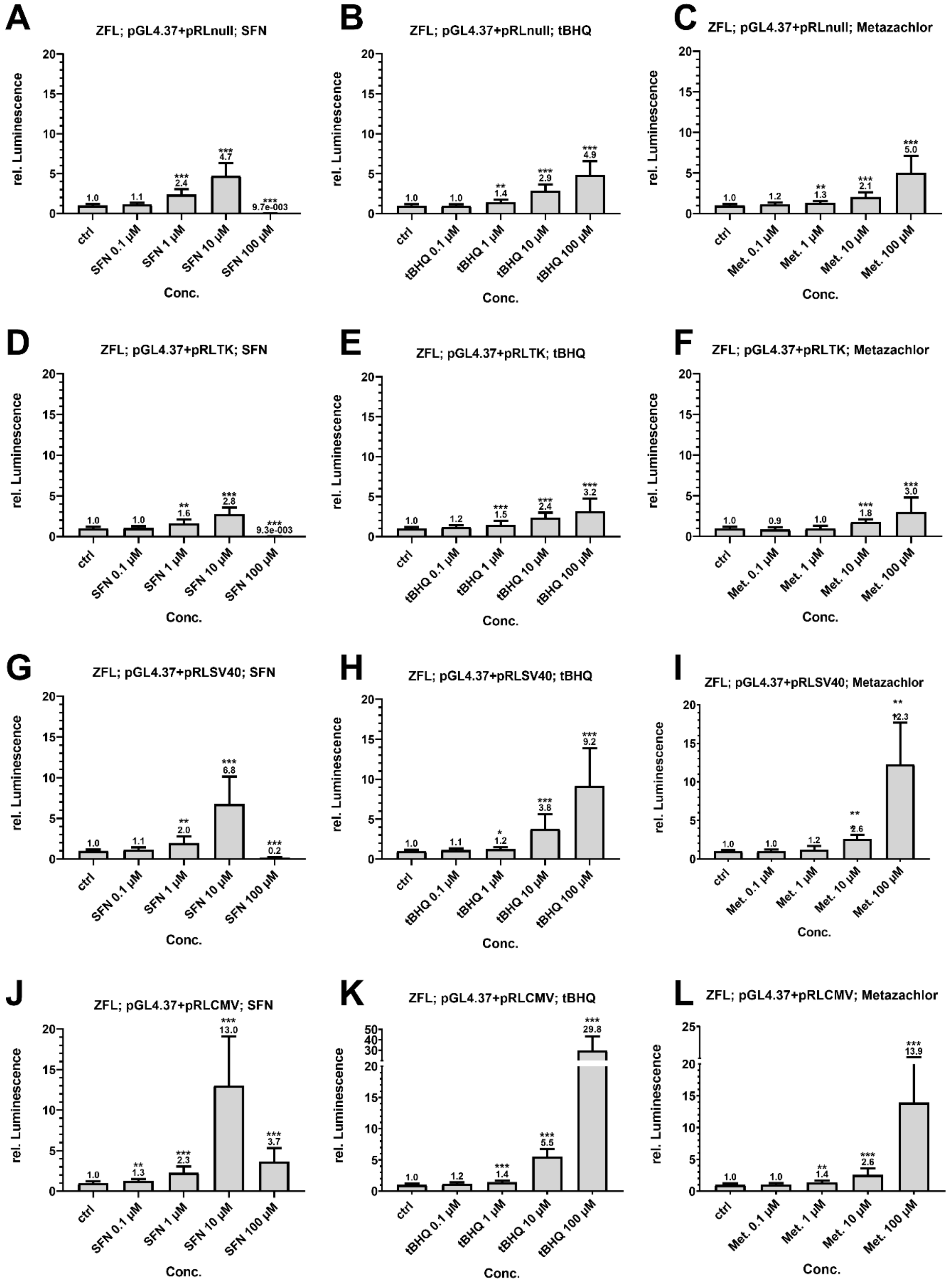

Fig. S3: Effects on normalized Nrf2 induction by promoters on the normalization vector in ZFL cells treated with sulforaphane (SFN; first column; A, D, G, J), *tert*butylhydroquinone (tBHQ, second column; B, E, H, K), and metazachlor (Met.; third column; C, F, I, L). Luminescence corresponds to quantitative Nrf2 activation measured via DLR assay in cells co-transfected with pGL4.37 and the respective pRLx normalization vector (null first row (A-C), TK second row (D-E), SV40 third row (G-I), CMV fourth row (J-L)). Each bar represents the mean (experimental units n = 3–4; observational units N = 10–16) including SD. Numerical means are depicted on top of bars. Asterisks indicate significance tested in a two-way ANOVA mixed model with Dunnett's post-hoc test (*$P < 0.05$, ** $P < 0.01$, *** $P < 0.001$).

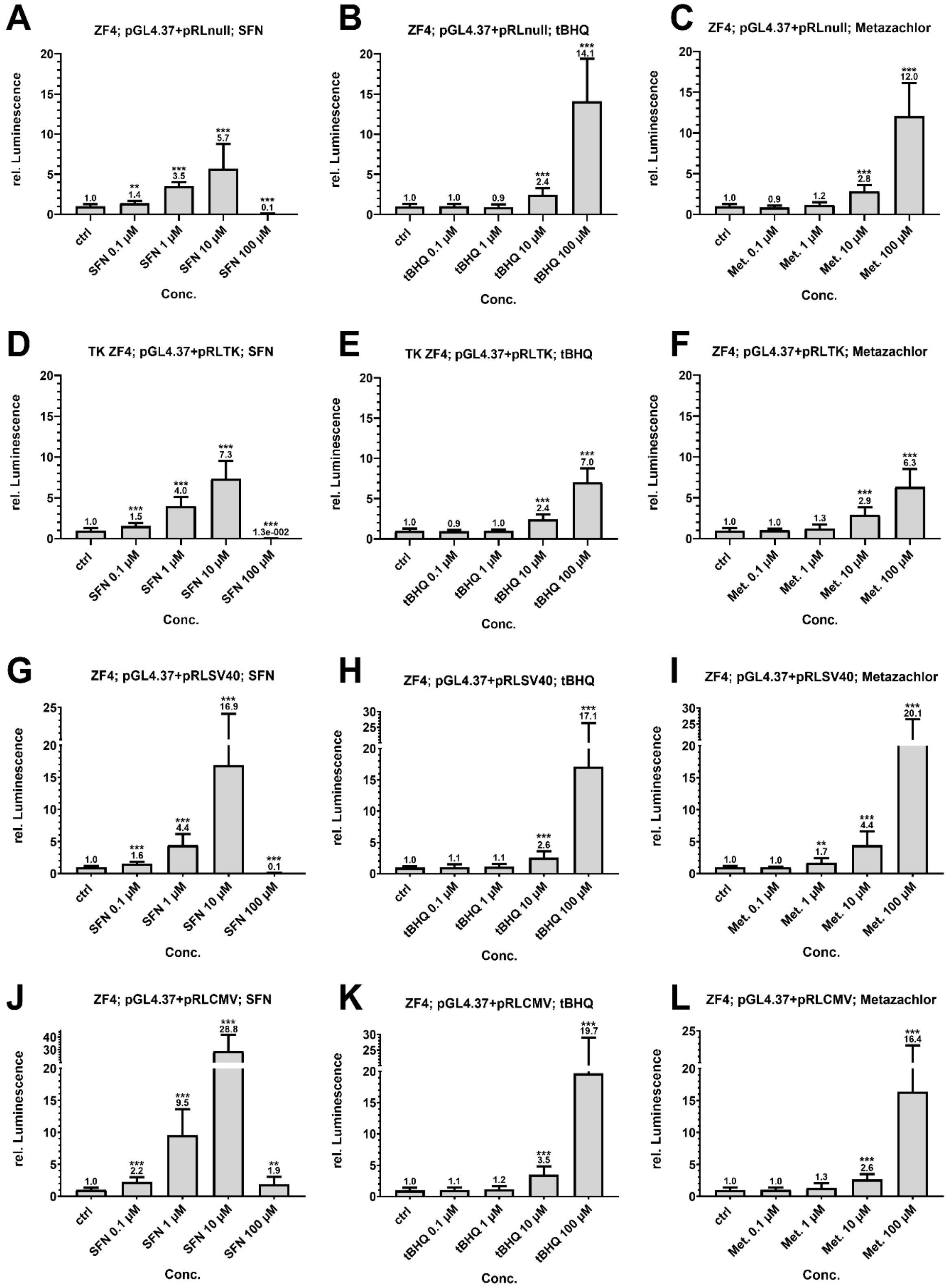

Fig. S4: Effects on normalized Nrf2 induction by promoters on the normalization vector in ZF4 cells treated with sulforaphane (SFN; first column; A, D, G, J), *tert*butylhydroquinone (tBHQ, second column; B, E, H, K), and metazachlor (Met.; third column; C, F, I, L). Luminescence corresponds to quantitative Nrf2 activation measured via DLR assay in cells co-transfected with pGL4.37 and the respective pRLx normalization vector (null first row (A-C), TK second row (D-E), SV40 third row (G-I), CMV fourth row (J-L)). Each bar represents the mean (experimental units n = 3–4; observational units N = 10–16) including SD. Numerical means are depicted on top of bars. Asterisks indicate significance tested in a two-way ANOVA mixed model with Dunnett's post-hoc test (**P* < 0.05, ** *P* < 0.01, *** *P* < 0.001).

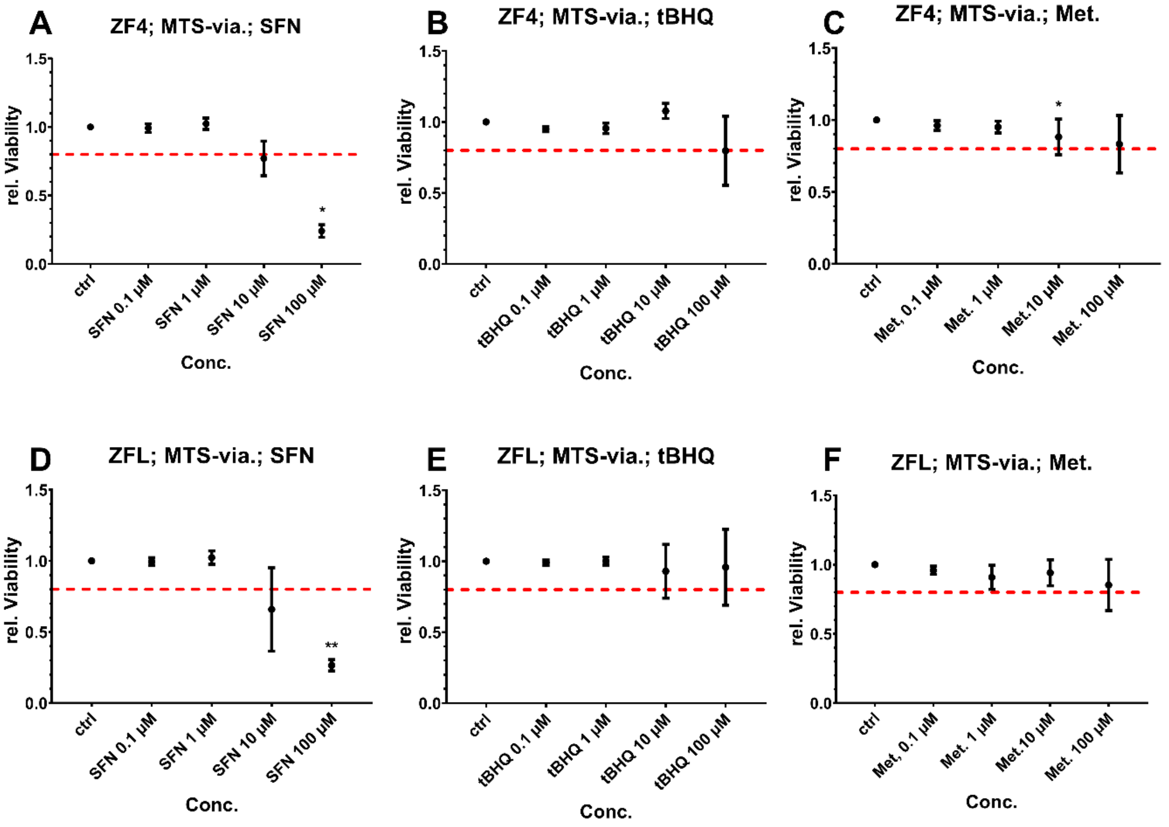

Fig. S5: Cell viability after treatment of ZF4 and ZFL cells with sulforaphane (SFN; first column; A+D), *tert*butylhydroquinone (tBHQ, second column, B+E), and metazachlor (Met.; third column; C+F). Viability corresponds to measured absorbance of formazan product via MTS-assay. A solvent control was used as the positive control (ctrl), to which the data was normalized. A threshold value of 0.8 was considered biologically relevant (dotted red line). Observational units (three replicates) were pooled, and means (experimental units n = 3-4), including SD, were analyzed. Asterisks indicate significance tested by Kruskal-Wallis Test with Dunn's post-hoc test (*$P < 0.05$, ** $P < 0.01$, *** $P < 0.001$).

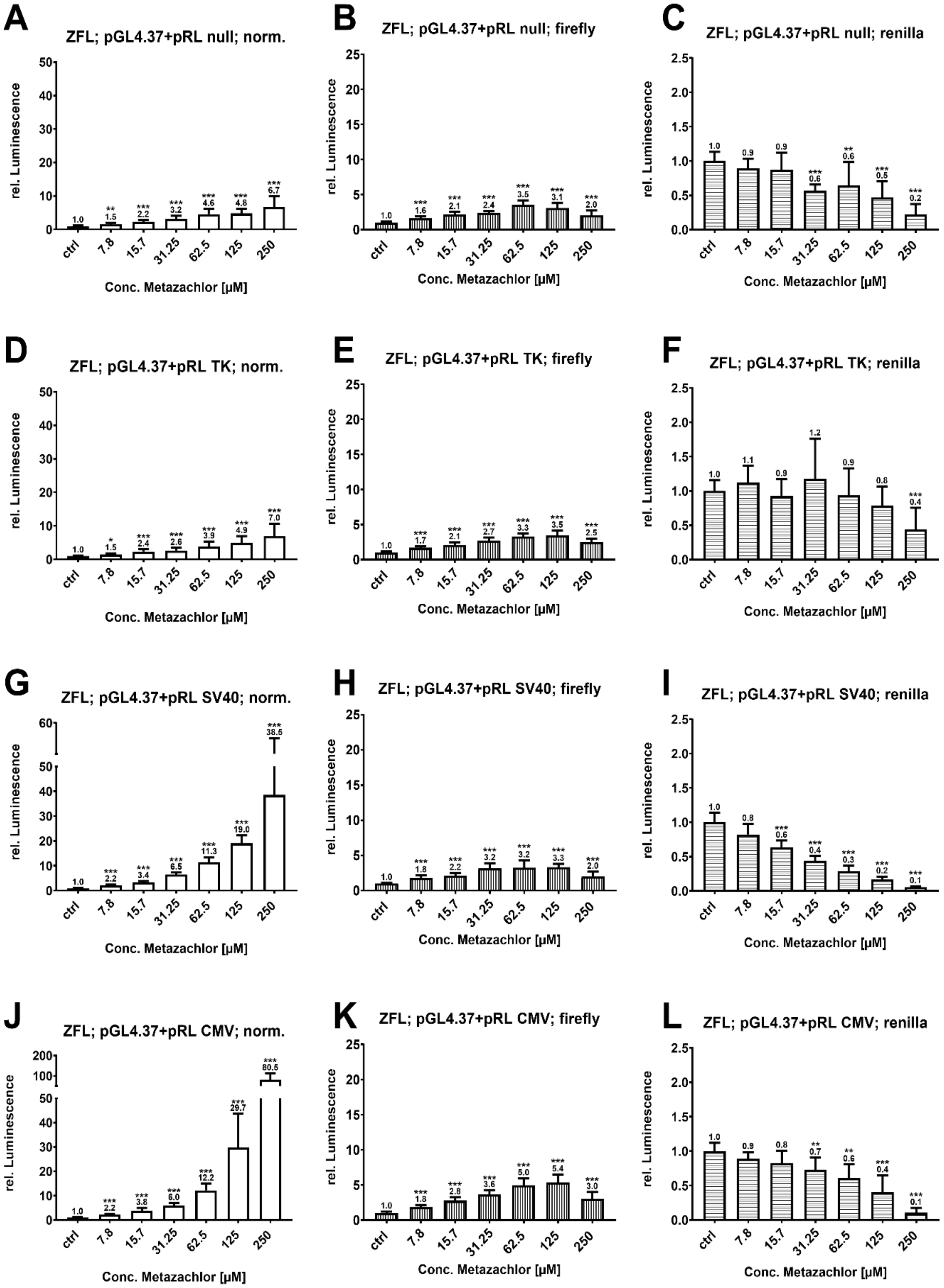

Fig. S6: Effects on luminescence measured in the zebrafish cell line ZFL treated with metazachlor (Met.). Luminescence corresponds to quantitative Nrf2 activation measured via DLR assay in cells co-transfected with pGL4.37 and the respective pRLx normalization vector (null first row (A-C), TK second row (D-E), SV40 third row (G-I), CMV fourth row (J-L)). Normalized values are depicted as white bars, Firefly luciferase read-outs as grey bars with vertical stripes, and Renilla luciferase read-outs as grey bars with horizontal stripes. Each bar represents the mean (experimental units n = 3–4; observational units N = 10–16) including SD. Numerical means are depicted on top of bars. Asterisks indicate significance tested in a two-way ANOVA mixed model with Dunnett's post-hoc test (*$P < 0.05$, ** $P < 0.01$, *** $P < 0.001$).

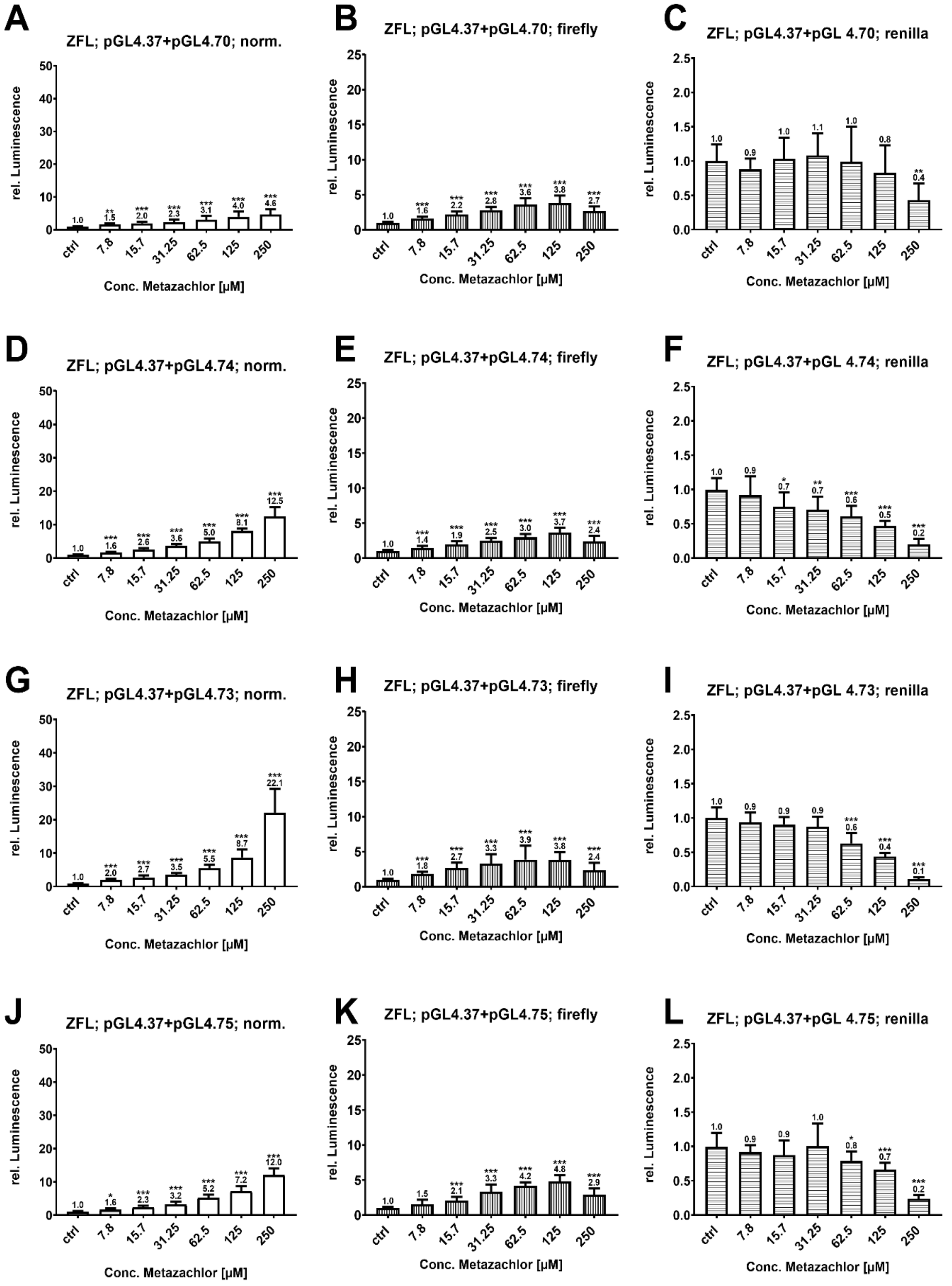

Fig. S7: Effects on luminescence measured in the zebrafish cell line ZFL treated with metazachlor (Met.). Luminescence corresponds to quantitative Nrf2 activation measured via DLR assay in cells co-transfected with pGL4.37 and the respective pGL4.7x normalization vector (null first row (A-C), TK second row (D-E), SV40 third row (G-I), CMV fourth row (J-L)). Normalized values are depicted as white bars, Firefly luciferase read-outs as grey bars with vertical stripes, and Renilla luciferase read-outs as grey bars with horizontal stripes. Each bar represents the mean (experimental units n = 3–4; observational units N = 10–16) including SD. Numerical means are depicted on top of bars. Asterisks indicate significance tested in a two-way ANOVA mixed model with Dunnett's post-hoc test (**P* < 0.05, ** *P* < 0.01, *** *P* < 0.001).

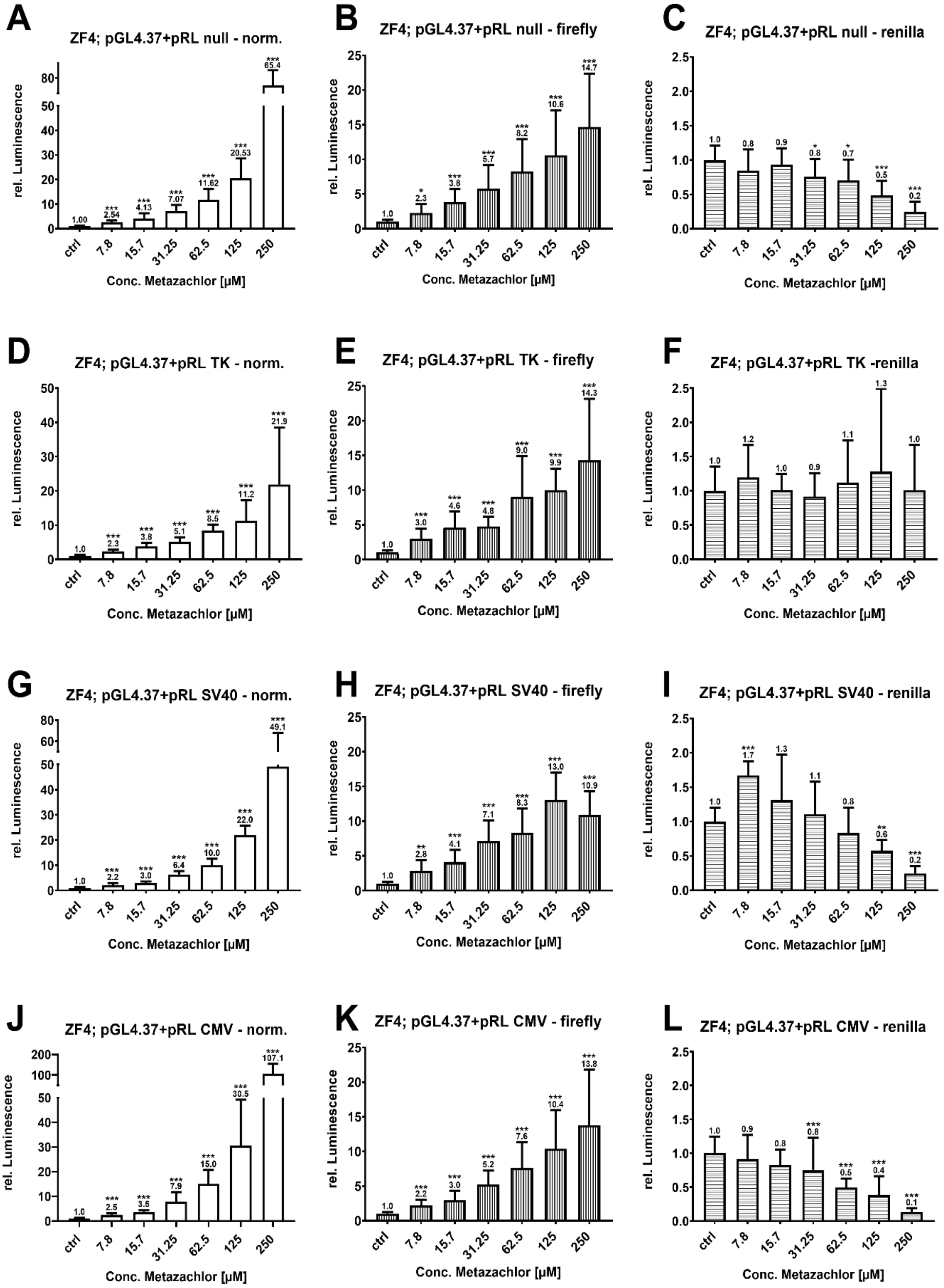

Fig. S8: Effects on luminescence measured in the zebrafish cell line ZF4 treated with metazachlor (Met.). Luminescence corresponds to quantitative Nrf2 activation measured via DLR assay in cells co-transfected with pGL4.37 and the respective pRLx normalization vector (null first row (A-C), TK second row (D-E), SV40 third row (G-I), CMV fourth row (J-L)). Normalized values are depicted as white bars, Firefly luciferase read-outs as grey bars with vertical stripes, and Renilla luciferase read-outs as grey bars with horizontal stripes. Each bar represents the mean (experimental units n = 3–4; observational units N = 10–16) including SD. Numerical means are depicted on top of bars. Asterisks indicate significance tested in a two-way ANOVA mixed model with Dunnett's post-hoc test (*$P < 0.05$, ** $P < 0.01$, *** $P < 0.001$).

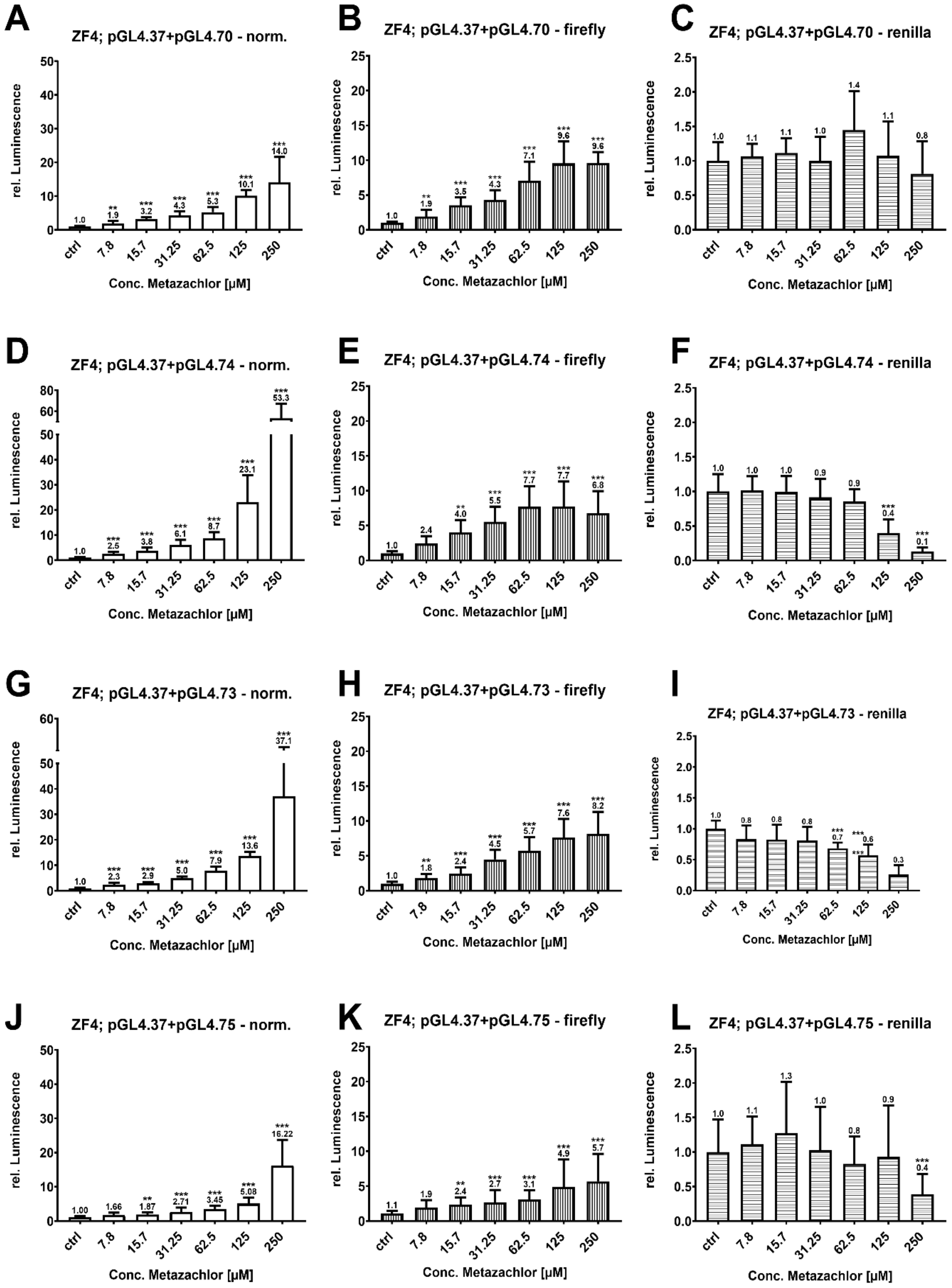

Fig. S9: Effects on luminescence measured in the zebrafish cell line ZF4 treated with metazachlor (Met.). Luminescence corresponds to quantitative Nrf2 activation measured via DLR assay in cells co-transfected with pGL4.37 and the respective pGL4.7x normalization vector (null first row (A-C), TK second row (D-E), SV40 third row (G-I), CMV fourth row (J-L)). Normalized values are depicted as white bars, Firefly luciferase read-outs as grey bars with vertical stripes, and Renilla luciferase read-outs as grey bars with horizontal stripes. Each bar represents the mean (experimental units n = 3–4; observational units N = 10–16) including SD. Numerical means are depicted on top of bars. Asterisks indicate significance tested in a two-way ANOVA mixed model with Dunnett's post-hoc test (*$P < 0.05$, ** $P < 0.01$, *** $P < 0.001$).

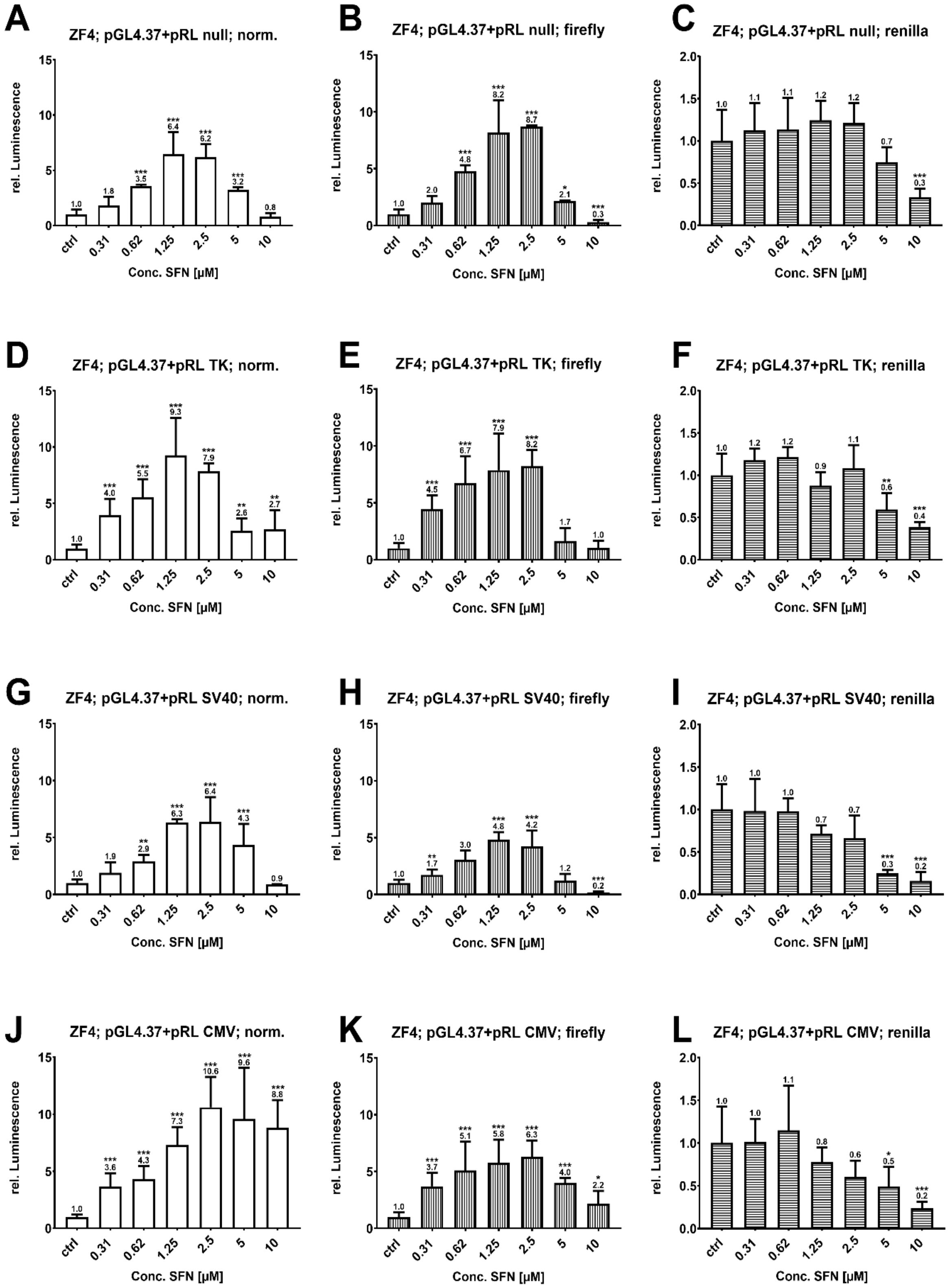

Fig. S10: Effects on luminescence measured in the zebrafish cell line ZF4 treated with sulforaphane (SFN). Luminescence corresponds to quantitative Nrf2 activation measured via DLR assay in cells co-transfected with pGL4.37 and the respective pRLx normalization vector (null first row (A-C), TK second row (D-E), SV40 third row (G-I), CMV fourth row (J-L)). Normalized values are depicted as white bars, Firefly luciferase read-outs as grey bars with vertical stripes, and Renilla luciferase read-outs as grey bars with horizontal stripes. Each bar represents the mean (experimental units n = 1; observational units N = 4) including SD. Numerical means are depicted on top of bars. Asterisks indicate significance tested in a one-way ANOVA linear model with Dunnett's post-hoc test (*$P < 0.05$, ** $P < 0.01$, *** $P < 0.001$).

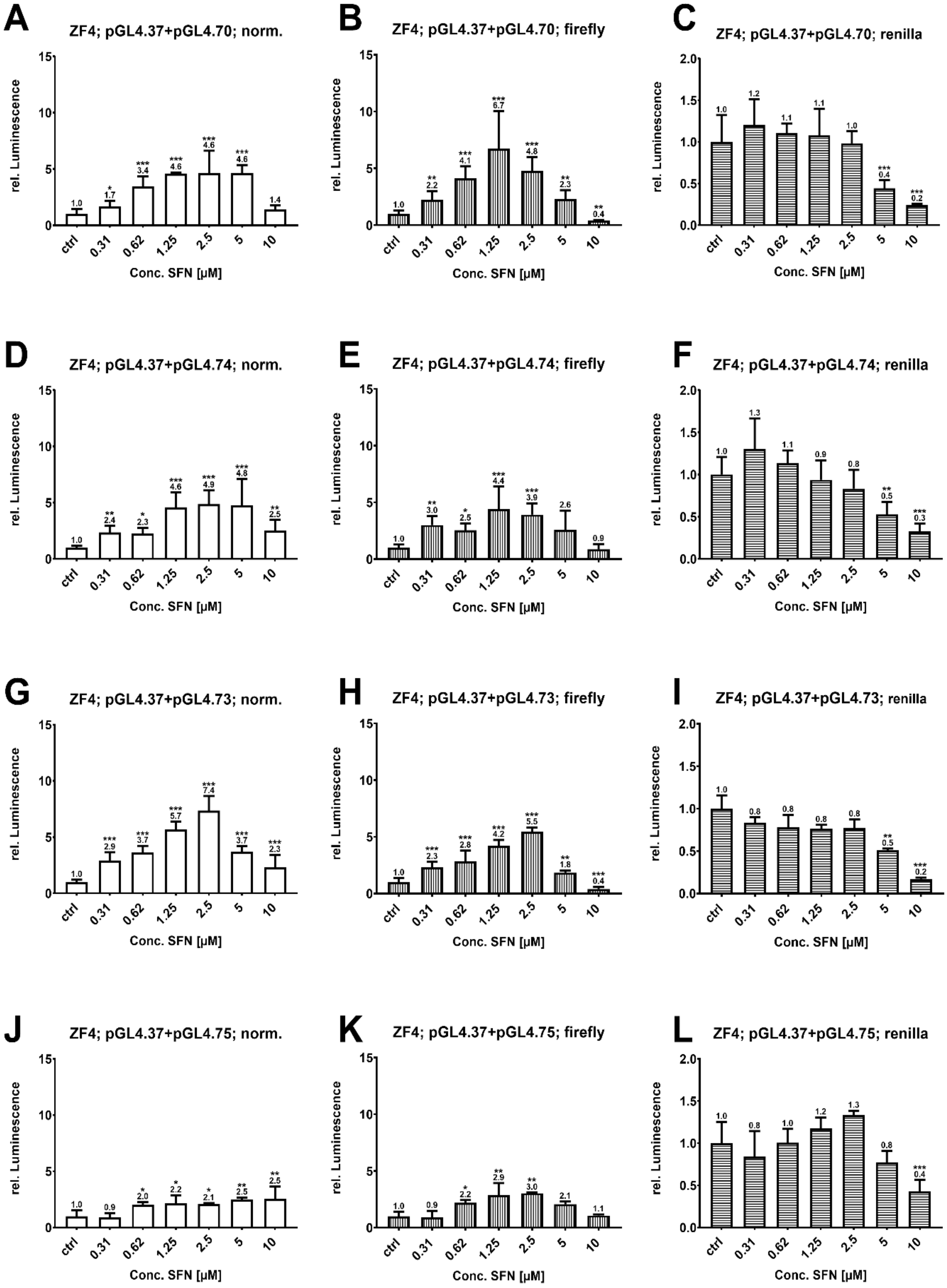

Fig. S11: Effects on luminescence measured in the zebrafish cell line ZF4 treated with sulforaphane (SFN). Luminescence corresponds to quantitative Nrf2 activation measured via DLR assay in cells co-transfected with pGL4.37 and the respective pGL4.7x normalization vector (null first row (A-C), TK second row (D-E), SV40 third row (G-I), CMV fourth row (J-L)). Normalized values are depicted as white bars, Firefly luciferase read-outs as grey bars with vertical stripes, and Renilla luciferase read-outs as grey bars with horizontal stripes. Each bar represents the mean (experimental units n = 1; observational units N = 4) including SD. Numerical means are depicted on top of bars. Asterisks indicate significance tested in a one-way ANOVA linear model with Dunnett's post-hoc test (*$P < 0.05$, ** $P < 0.01$, *** $P < 0.001$).

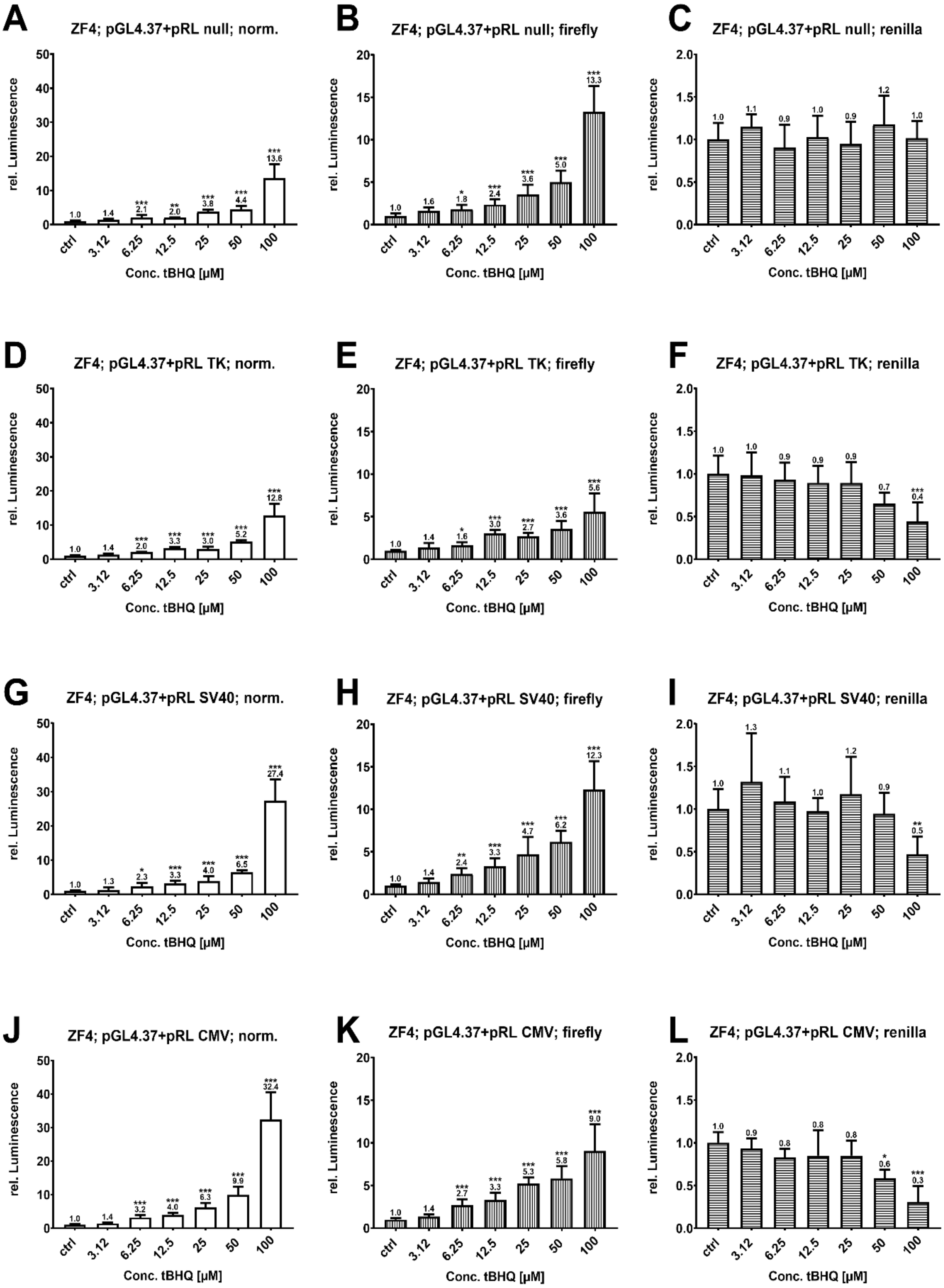

Fig. S12: Effects on luminescence measured in the zebrafish cell line ZF4 treated with *tert*butylhydroquinone (tBHQ). Luminescence corresponds to quantitative Nrf2 activation measured via DLR assay in cells co-transfected with pGL4.37 and the respective pRLx normalization vector (null first row (A-C), TK second row (D-E), SV40 third row (G-I), CMV fourth row (J-L)). Normalized values are depicted as white bars, Firefly luciferase read-outs as grey bars with vertical stripes, and Renilla luciferase read-outs as grey bars with horizontal stripes. Each bar represents the mean (experimental units n = 1; observational units N = 4) including SD. Numerical means are depicted on top of bars. Asterisks indicate significance tested in a one-way ANOVA linear model with Dunnett's post-hoc test (*$P < 0.05$, ** $P < 0.01$, *** $P < 0.001$).

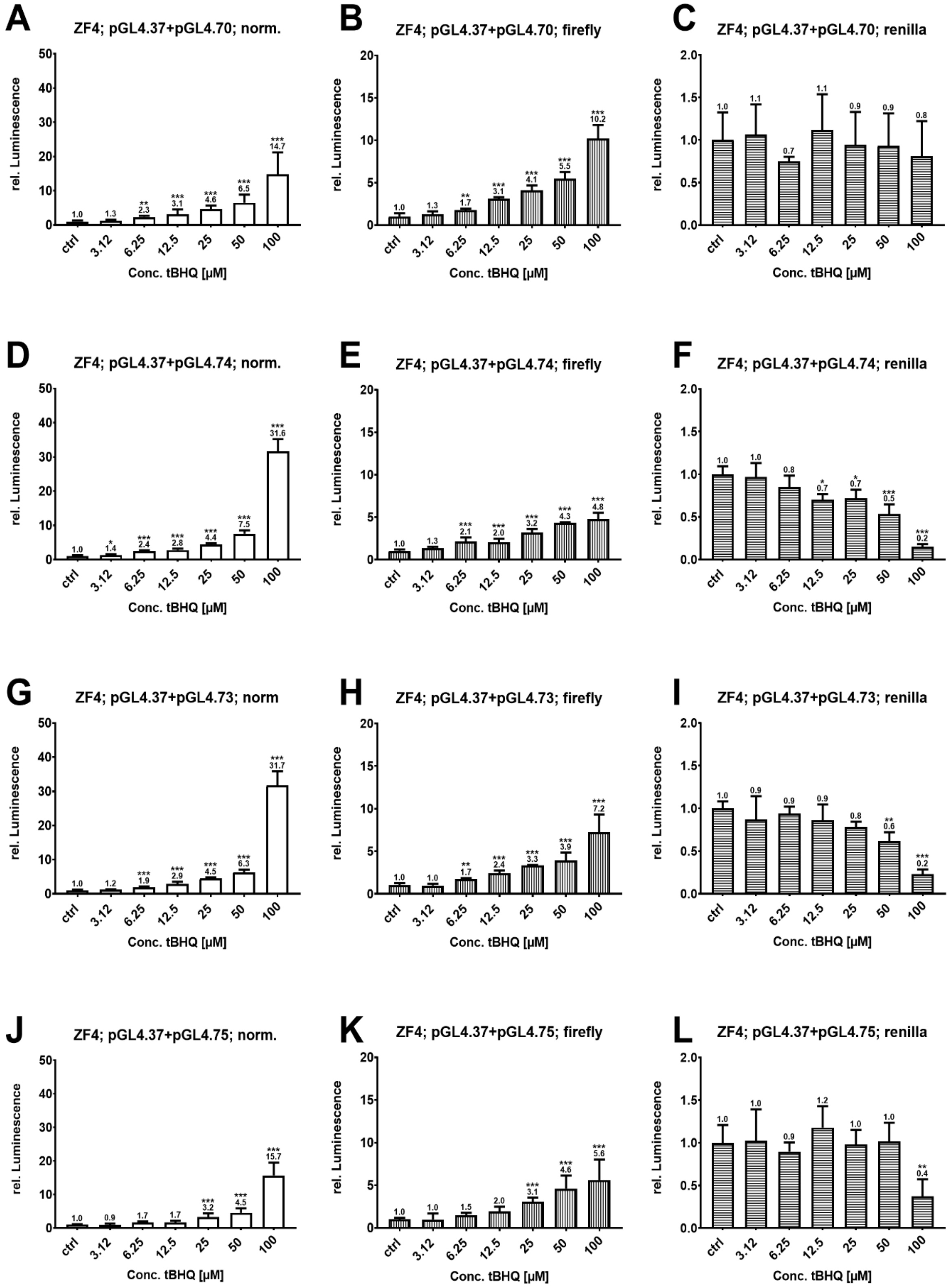

Fig. S13: Effects on luminescence measured in the zebrafish cell line ZF4 treated with *tert*butylhydroquinone (tBHQ). Luminescence corresponds to quantitative Nrf2 activation measured via DLR assay in cells co-transfected with pGL4.37 and the respective pGL4x normalization vector (null first row (A-C), TK second row (D-E), SV40 third row (G-I), CMV fourth row (J-L)). Normalized values are depicted as white bars, Firefly luciferase read-outs as grey bars with vertical stripes, and Renilla luciferase read-outs as grey bars with horizontal stripes. Each bar represents the mean (experimental units n = 1; observational units N = 4) including SD. Numerical means are depicted on top of bars. Asterisks indicate significance tested in a one-way ANOVA linear model with Dunnett's post-hoc test (**P* < 0.05, ** *P* < 0.01, *** *P* < 0.001).

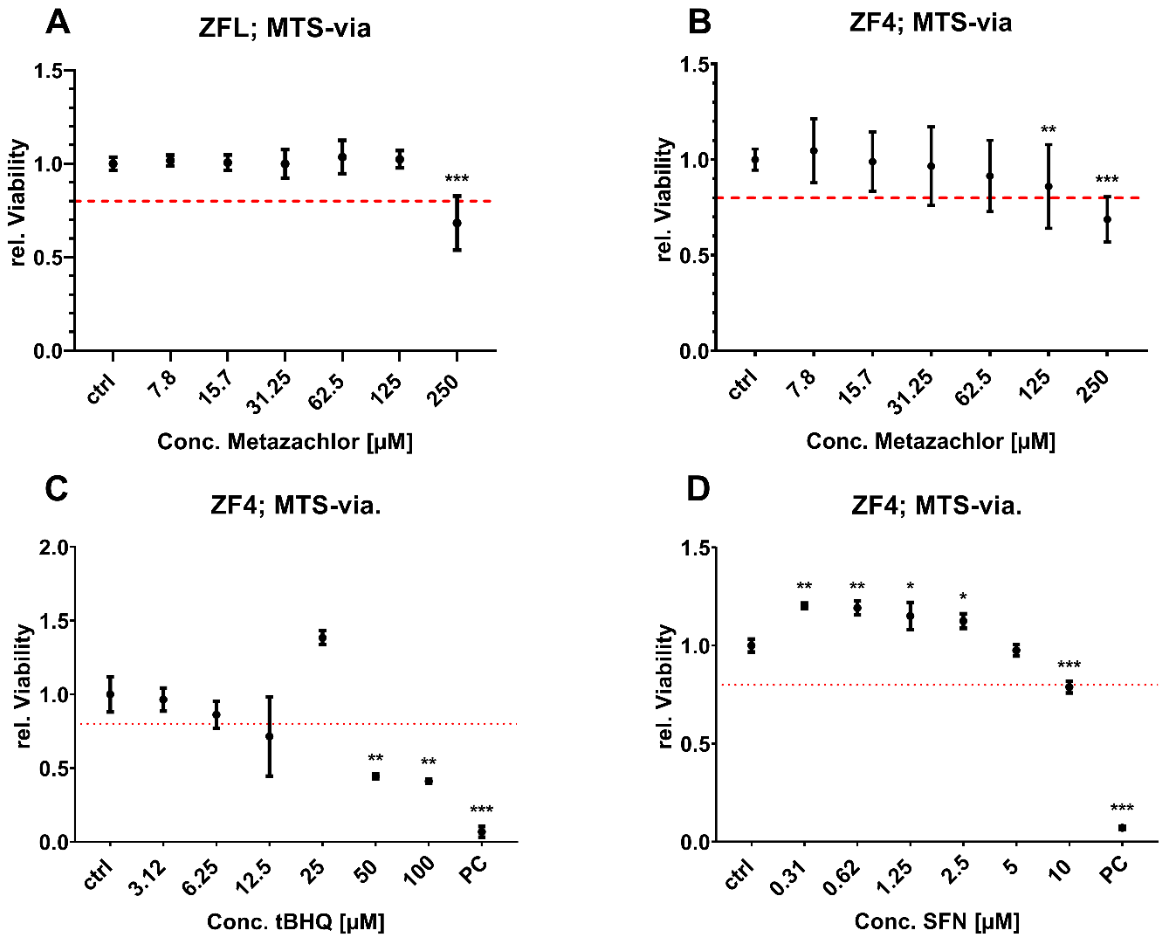

Fig. S14: Cell viability after treatment of ZFL (A) and ZF4 (B) cells with metazachlor. Viability corresponds to measured absorbance of formazan product via MTS-assay. A solvent control was used as the positive control (ctrl), to which the data was normalized. A threshold value of 0.8 was considered biologically relevant (dotted red line). Each point represents the mean (experimental units n = 3–4; observational units N = 9-12) including SD. Asterisks indicate significance tested in a two-way ANOVA mixed model with Dunnett's post-hoc test (*p < 0.05, **p < 0.01, ***p < 0.001). Effects on viability after treatment of ZF4 cells to *tert*butylhydroquinone (tBHQ; C) and sulforaphane (SFN; D). 10% (v/v) of DMSO in nutrition medium were used as positive controls (PC). Each point represents the mean (experimental units n = 1; observational units N = 3) including SD. Asterisks indicate significance tested in a one-way ANOVA linear model with Dunnett's post-hoc test (**P* < 0.05, ** *P* < 0.01, *** *P* < 0.001).

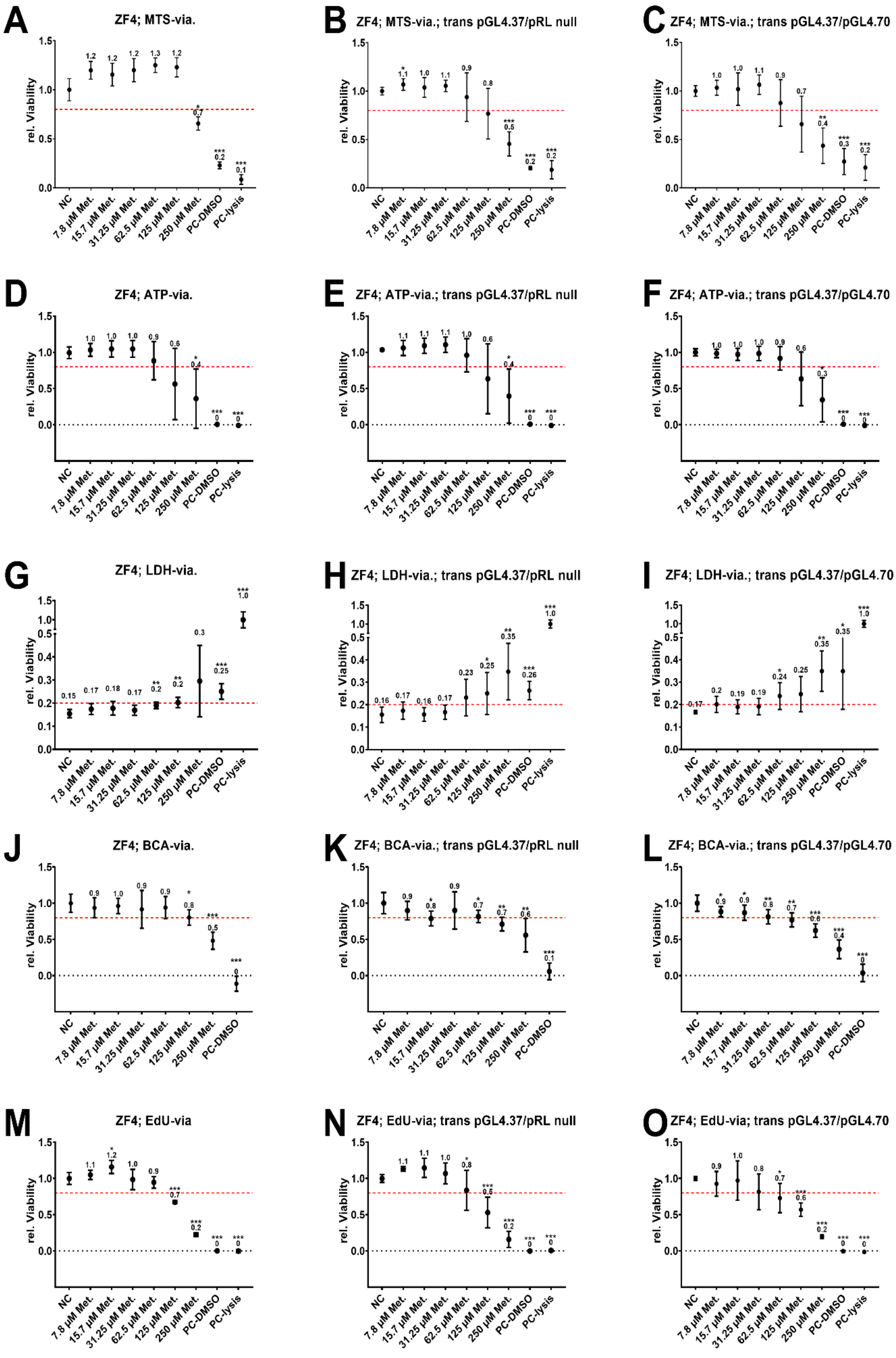

A
ZF4; MTS-via.
B
ZF4; MTS-via.; trans pGL4.37/pRL null
C
ZF4; MTS-via.; trans pGL4.37/pGL4.70
D
ZF4; ATP-via.
E
ZF4; ATP-via.; trans pGL4.37/pRL null
F
ZF4; ATP-via.; trans pGL4.37/pGL4.70
G
ZF4; LDH-via.
H
ZF4; LDH-via.; trans pGL4.37/pRL null
I
ZF4; LDH-via.; trans pGL4.37/pGL4.70
J
ZF4; BCA-via.
K
ZF4; BCA-via.; trans pGL4.37/pRL null
L
ZF4; BCA-via.; trans pGL4.37/pGL4.70
M
ZF4; EdU-via
N
ZF4; EdU-via; trans pGL4.37/pRL null
O
ZF4; EdU-via; trans pGL4.37/pGL4.70
rel. Viability
NC
7.8 µM Met.
15.7 µM Met.
31.25 µM Met.
62.5 µM Met.
125 µM Met.
250 µM Met.
PC-DMSO
PC-lysis

Fig. S15: Effects on various viability endpoints (dots) measured in the zebrafish cell line ZF4 treated with metazachlor (Met.). Endpoints quantified are NAPDH metabolism via the MTS-assay (A-C), ATP turnover (D-F) and LDH release (G-I) via the ATP/LDH multiplex assay, total protein amount via the BCA assay (J-L), and cell proliferation via the EdU-assay (M-O). A solvent control was used as negative control (NC). Cellular lysis buffer (PC-lysis) and 10% (v/v) of DMSO in nutrition medium (PC-DMSO) were used as positive controls. Initial values were normalized to the NC or PC (G-I), respectively. A threshold value of 0.8 or 0.2 (for LDH) was considered biologically relevant (dotted red line). Each point represents the mean (experimental units n = 3–4; observational units N = 9-12) including SD. Asterisks indicate significance tested in a two-way ANOVA mixed model with Dunnett's post-hoc test (*$P < 0.05$, ** $P < 0.01$, *** $P < 0.001$). For every endpoint, firstly non-transfected cells were exposed (first column; A, D, G, J, M) and secondly, cells were co-transfected with pGL4.37 and normalization vectors of increasing size, pRL null (3320 nt; second column; B, E, H, K, N) and pGL4.70 (3522 nt; third column, C, F, I, L, O).

Tab. S6: Threshold-based (tre.) and statistically (stat.) derived LOECs in [µM] after metazachlor exposure in ZF4 cells using assays for diverse cellular viability endpoints, relative inhibition values are given in parenthesis; for respective graphical illustrations see fig. 4 and fig. S15.

	**Non-transfected**	**Trans. pGL4.37/pRL null**	**Trans. pGL4.37/pGL4.70**
**MTS-assay**	250 tre. (0.7)	125 tre. (0.8)	125 tre. (0.7)
(fig. S15 A-C)	250 stat. (0.7)	250 stat. (0.5)	250 stat. (0.4)
**ATP-assay**	125 tre. (0.6)	125 tre. (0.6)	125 tre. (0.6)
(fig. S15 D-F)	250 stat. (0.4)	250 stat. (0.4)	250 stat. (0.3)
**LDH-assay**	62.5 tre. (0.2)	62.5 tre. (0.23)	62.5 tre. (0.24)
(fig. S15 G-I)	62.5 stat. (0.2)	125 stat. (0.25)	62.5 stat. (0.24)
**BCA-assay**	125 tre. (0.8)	15.7 tre. (0.8)	31.25 tre. (0.8)
(fig. S15 J-L)	125 stat. (0.8)	15.7 stat. (0.8)	7.8 stat. (0.9)
**EdU-assay**	125 tre. (0.7)	62.5 tre. (0.8)	31.25 tre. (0.8)
(fig. S15 M-O)	125 stat. (0.7)	62.5 stat. (0.8)	62.5 tre. (0.7)
